# Haematology and blood chemistry in free-ranging quokkas (Setonix brachyurus): Reference intervals and assessing the effects of site, sampling time, and infectious agents

**DOI:** 10.1371/journal.pone.0239060

**Published:** 2020-09-17

**Authors:** Pedro A. Martínez-Pérez, Timothy H. Hyndman, Patricia A. Fleming

**Affiliations:** 1 School of Veterinary Medicine, Murdoch University, Murdoch, Western Australia, Australia; 2 Harry Butler Institute, Murdoch University, Murdoch, Western Australia, Australia; Leibniz Institute for Zoo and Wildlife Research (IZW), GERMANY

## Abstract

Quokkas (*Setonix brachyurus*) are small macropodid marsupials from Western Australia, which are identified as of conservation concern. Studies on their blood analytes exist but involve small sample sizes and are associated with very little information concerning the health of the animals. Blood was collected from free-ranging quokkas from Rottnest Island (n = 113) and mainland (n = 37) Western Australia, between September 2010 and December 2011, to establish haematology and blood chemistry reference intervals. Differences in haematology and blood chemistry between sites (Rottnest Island v mainland) were significant for haematology (HMT, *p* = 0.003), blood chemistry (BLC, *p* = 0.001) and peripheral blood cell morphology (PBCM, *p* = 0.001). Except for alkaline phosphatase, all blood chemistry analytes were higher in mainland animals. There were also differences with time of year in HMT (*p* = 0.001), BLC (*p* = 0.001) and PBCM (*p* = 0.001) for Rottnest Island quokkas. A small sample of captive animals (n = 8) were opportunistically sampled for plasma concentrations of vitamin E and were found to be deficient compared with wild-caught animals. Fifty-eight of the 150 quokkas were also tested for the presence of *Salmonella*, microfilariae, *Macropodid herpesvirus-6*, *Theileria* spp., *Babesia* spp., trypanosomes, *Cryptococcus* spp. and other saprophytic fungi. All eight infectious agents were detected in this study. Infectious agents were detected in 24 of these 58 quokkas (41%), with more than one infectious agent detected for all 24 individuals. *Salmonella* were detected concurrently with microfilariae in 8 of these 24 quokkas, and this mixed infection was associated with lower values across all haematological analytes, with *Salmonella* having the greater involvement in the decreased haematological values (*p* < 0.05). There was no evidence for an effect of sex on HMT, BLC and PBCM. Our data provide important haematological and blood chemistry reference intervals for free-ranging quokkas. We applied novel methods of analyses to HMT and BLC that can be used more broadly, aiding identification of potential disease in wildlife.

## Introduction

The quokka is a small macropod endemic to Western Australia [1]. These marsupials have a restricted distribution and the species is recognised as vulnerable under the International Union for Conservation of Nature Red List [2]. Quokkas are found in relative abundance on Rottnest Island (~20 km^2^), which is also a tourism destination, attracting ~750,000 visitors annually (2018 data). In contrast, populations on the mainland live in forested areas and are rare and elusive [1].

Blood analysis is a common tool for assessment of animal health. Blood analyses can assist with diagnosis of disease, establish the impact of a disease, or determine the response to therapy. However, the use of blood test results is compromised by the absence of baseline data against which the degree of change (from "normal") can be assessed. For example, the absence of reference intervals hindered the interpretation of haematological test results in response to a novel papillomatosis and carcinomatosis syndrome in western barred bandicoots (*Perameles bougainville*) [3]. Even where they are available, reported reference intervals are frequently obtained from small populations or populations in captivity, often with little supplementary information available to help determine if this population should be providing reference intervals that will be defined as "normal" for that species [4, 5]. Prompt and educated responses in the event of a disease outbreak is therefore facilitated by the existence of data on the haematology (HMT) and blood chemistry (BLC) values of apparently healthy individuals of a particular species.

While studies do exist on the HMT [6–15] and BLC [10, 15] in quokkas, this has scarcely been for free-ranging populations [6–8] and mostly for captive animals that are likely to have quite different values for at least some parameters, e.g. due to different nutrition [9–15]. From these studies, only three report white blood cell data (i.e. white blood cell count, differential counts) [11, 13, 14] and only one reports BLC data from 2–4 (depending on the BLC analyte) captive quokkas [15].

In this study, we examined the differences in the HMT and BLC profiles of free-ranging quokkas from Rottnest Island and the mainland of WA. We also examined the influence that site (Rottnest Island v mainland), time of year (Rottnest Island animals only), sex and the presence or absence of infectious agents had on these profiles. Using this information, we constructed reference intervals for apparently-healthy free-ranging quokkas for both sites.

## Materials and methods

### Animal ethics statement

All animal work was carried out under permits from the Murdoch University Animal Ethics Committee (W2309/10) and the Department of Biodiversity, Conservation and Attractions (SF007550 and CE002891).

### Sample collection and testing

Between September 2010 and December 2011, quokkas were captured on Rottnest Island and on the mainland in southwest Western Australia. On Rottnest Island, Thomas® traps (Sheffield Wire Products, Australia) were deployed after sunset, baited with peanut butter and oats, and cleared every hour until midnight. On the mainland, Sheffield® traps (Sheffield Wire Products, Australia) were deployed after sunset, set along water systems, baited with apples, and cleared between 06:00–09:00h the following morning. On Rottnest Island, samples were collected over four separate timepoints that were similar to the Austral seasons. As each season was only sampled once, we could not infer any seasonal effects. However, for convenience, we refer to these sample times by season in some figures. On the mainland, samples were collected opportunistically throughout the year to fit in with trapping for other studies [16, 17]. A small sample of captive, apparently-healthy adults belonging to Perth Zoo (n = 8; originally sourced from Rottnest Island) were opportunistically sampled for vitamin E only; their diet is described in S1 File. Animals were removed from traps (excluding Perth Zoo quokkas) and transferred in hessian handling bags to a field setup for clinical examination under anaesthesia, induced with 5% isoflurane (I.S.O.®, Veterinary Companies of Australia, Australia) delivered in 100% medical oxygen (2.5 L/min). Anaesthesia was maintained at 2–3% isoflurane and 2 L/min of oxygen. While anaesthetised, samples were collected and a thorough physical examination was performed on each animal, including an assessment of its body condition on a scale of 1–5 (S1 Table).

#### Blood samples

Blood samples were collected from the lateral tail vein using Safety-Lok™ BD Vacutainer® with either a 25G x ¾” or 23G x ¾” needle with Slip Tip 3 mL syringes (Becton, Dickinson and Company, NJ, USA). Blood for blood chemistry (~1 mL) analyses were collected in 1.3 mL Micro Tubes with 35 I.U. of lithium heparin (SARSTEDT, Aktiengesellschaft & Co. Nümbrecht, Germany), while samples for haematological analyses (~0.5 mL) were collected in 600 μL BD Microtainer® tubes with potassium EDTA (Becton, Dickinson and Company, NJ, USA). These two blood samples were mixed gently upon collection and stored at 4°C until further processing. Blood samples were submitted within 96 h of collection to the Clinical Pathology service of the Murdoch University Veterinary Hospital for haematology and blood chemistry analyses. Peripheral blood smears were made using the spreader slide technique and air-dried for storage.

#### Nasal swabs and faecal samples

For nasal swabs, the MiniTip Aluminium Wire Venturi Transystem® was used (Copan Italia S.p.A, Brescia, Italy). Each nostril of each animal was sampled by a single swab (i.e. two swabs per animal). This was done by gently introducing the swab 1.5–2 cm into the nasal vestibule and rotating it at least 10 times against the nasal lining. For faecal pellets, the external area around the cloaca was first cleaned with a 1:1 mixture of chlorhexidine gluconate and 70% ethanol. Faecal samples were retrieved through rectal palpation and then placed into 5 mL polycarbonate yellow cap sterile tubes (SARSTEDT Aktiengeseilschaft & Co. Germany) and stored at 4 ˚C until processing.

#### Screening tests for infectious agents

Blood samples were screened by PCR for the detection of *Macropodid herpesvirus-6*, *Theileria* spp., *Babesia* spp., and trypanosomes. All PCR-positives were sequenced. For *Macropodid herpesvirus-6*, a broadly-reactive PCR was used to detect a segment of the polymerase gene of members of the order *Herpesvirales* [18]. *Equine herpesvirus 1* was used as a positive control. Nested PCR was used to target the rRNA genes of trypanosomes [19] and piroplasms (*Babesia* spp. and *Theileria* spp.) [20]. Positive controls for these protozoa were kindly provided by Professor Una Ryan (Murdoch University).

Blood samples (EDTA) were visually screened for microfilariae as described below. For *Cryptococcus* spp. and other saprophytic fungi, nasal swabs were cultured onto bird seed agar and colonies were sub-cultured onto Sabouraud dextrose agar plates according to methods previously described [21]. Isolates were speciated using PCR and sequencing of a segment of the internal transcribed spacer region of the fungal genome [22]. For *Salmonella* spp., faecal pellets were pre-enriched in peptone water, secondarily enriched in Rappaport Vassiliadis selective enrichment broth, cultured onto xylose lysine deoxycholate agar plates and then sub-cultured onto nutrient agar plates before Gram's staining; biochemical testing and antiserum agglutination testing were used to identify the species and serovar of *Salmonella* spp. [23].

#### Haematology (HMT)

A complete blood count and erythrocyte indices (Table 1) were obtained with an ADVIA-120® automated haematology analyser (Bayer diagnostics division, Tarrytown, New York, USA) and multi-species software using the default setting (canine). We report Corpuscular Haemoglobin Concentration Mean (CHCM) instead of Mean Corpuscular Haemoglobin Concentration (MCHC), given that the former is obtained through laser-detection technology that allows for direct determination of haemoglobin in each erythrocyte, therefore not being affected by blood abnormalities that could increase the spectrophotometric reading (e.g. lipaemia, haemolysis) [24] which would be the case of MCHC (calculated).

**Table 1:** SIMPER analysis indicating the percent contribution (Ct%) of specific variables to the observed *site* [Rottnest Island (RI) and mainland (ML)] differences in (a) Haematology (HMT), (b) Blood chemistry (BLC), and (c) Peripheral blood cell morphology (PBCM) profiles of quokkas. For (a and b), mean with standard deviation. For (c), the number of animals (percentage of sampled animals) with 95% confidence intervals (§).

			RI		ML	
Analyte		Ct %	$\bar{x}$	SD	$\bar{x}$	SD
a) Haematology (HMT) (27.64*) RI n = 96, ML n = 32						
Red Blood Cell Conc. (x10^12^/L)	RBC	10.7	5.84	0.87	7.38	1.01
Lymphocytes (x10^9^/L)	LYMPH	10.1	2.09	1.09	1.64	1.08
Eosinophils (x10^9^/L)	EOS	9.86	0.40	0.365	0.14	0.214
Neutrophils (x10^9^/L)	NEUT	9.86	2.23	0.913	2.92	1.52
Packed Cell Volume (%)	PCV	9.61	33.4	4.46	40.9	4.08
Basophils (x10^9^/L)	BASO	9.32	0.02	0.03	0.03	0.03
Haemoglobin Conc. (g/L)	HGB	9.06	108	15.3	134	15.5
Monocytes (x10^9^/L)	MONO	8.79	0.08	0.08	0.08	0.07
White Blood Cell Count (x10^9^/L)	WBC	8.24	4.82	1.58	4.78	1.40
Mean Corpuscular Volume (fL)	MCV	7.43	61	3.65	59.8	3.27
Corpuscular Haemoglobin Conc. Mean (g/L)	CHCM	7.01	307	15.5	326	27.2
b) Blood chemistry (BLC) (22.76*) RI n = 106, ML n = 32						
Creatine Kinase (U/L)	CK	11.2	942	1,331	7,665	6,799
Alanine Aminotransferase (U/L)	ALT	9.80	217	62.2	436	156
Vitamin E (mg/L)	Vit. E	9.10	6.53	1.80	9.93	2.73
Alkaline Phosphatase (U/L)	ALP	7.62	8,204	10,094	8,074	13,947
Aspartate Aminotransferase (U/L)	AST	7.03	49.8	31.6	239	308
Phosphorus (mmol/L)	PHOSP	6.63	1.19	0.467	1.68	0.67
Total Protein (g/L)	TP	6.62	60.4	4.62	63.9	4.35
Calcium (mmol/L)	CALC	6.35	2.20	0.201	2.47	0.141
Glucose (mmol/L)	GLUC	6.07	4.15	2.21	5.46	2.33
Cholesterol (mmol/L)	CHOL	6.00	2.80	0.539	2.99	0.65
Albumin (g/L)	ALB	5.76	36.3	1.91	38.9	2.19
Total Bilirubin (μmol/L)	BILT	5.26	4.29	1.65	5.32	2.75
Globulin (g/L)	GLOB	5.04	24.1	3.75	24.9	3.56
Urea (mmol/L)	UREA	4.43	6.87	1.53	9.60	4.94
Creatinine (μmol/L)	CREAT	3.16	70.9	16	83.3	23.7
Gamma-glutamyl Transferase †	GGT					
c) Peripheral blood cell morphology (PBCM) (29.01*) RI n = 107, ML n = 34						
			n (%)	CI	n (%)	CI
Heinz Bodies		11.4	75 (70.1%)	0.61–0.78	20 (59%)	0.42–0.74
Acanthocytes		11	52 (49.0%)	0.39–0.58	19 (56%)	0.39–0.71
Rouleaux Formation		10.9	46 (43%)	0.34–0.52	17 (50%)	0.34–0.66
Hypochromasia		10.3	96 (90%)	0.83–0.94	19 (56%)	0.39–0.71
Nucleated red blood cells	nRBCs	8.04	97 (91%)	0.84–0.95	23 (68%)	0.51–0.81
Anisocytosis		8.03	86 (80%)	0.72–0.87	25 (73.5%)	0.57–0.85
Echinocytes		7.88	30 (28%)	0.20–0.37	7 (21%)	0.10–0.37
Polychromasia		7.51	98 (92%)	0.85–0.96	24 (71%)	0.54–0.83
Schistocytes		6.40	26 (24.3%)	0.17–0.33	4 (12%)	0.05–0.27
Poikilocytosis		5.45	93 (87%)	0.80–0.92	29 (85%)	0.70–0.94
Keratocytes		4.91	24 (22.4%)	0.15–031	1 (3%)	0.01–0.15
Flower Cells		4.52	23 (21.5%)	0.14–0.30	0 (0%)	0.00–0.10
Howell-Jolly Bodies		3.42	99 (92.5%)	0.86–0.96	31 (91%)	0.77–0.97
Reactive Lymphocytes		0.29	106 (99.1%)	0.96–0.99	34 (100%)	0.89–1.00

* Overall average dissimilarity.

§ Confidence intervals calculated using Jeffreys model [25] for RI or using Wilson’s model [25] for ML.

† included only for reference intervals due to sample size restrictions for multivariate analyses.

**Fig 1:**
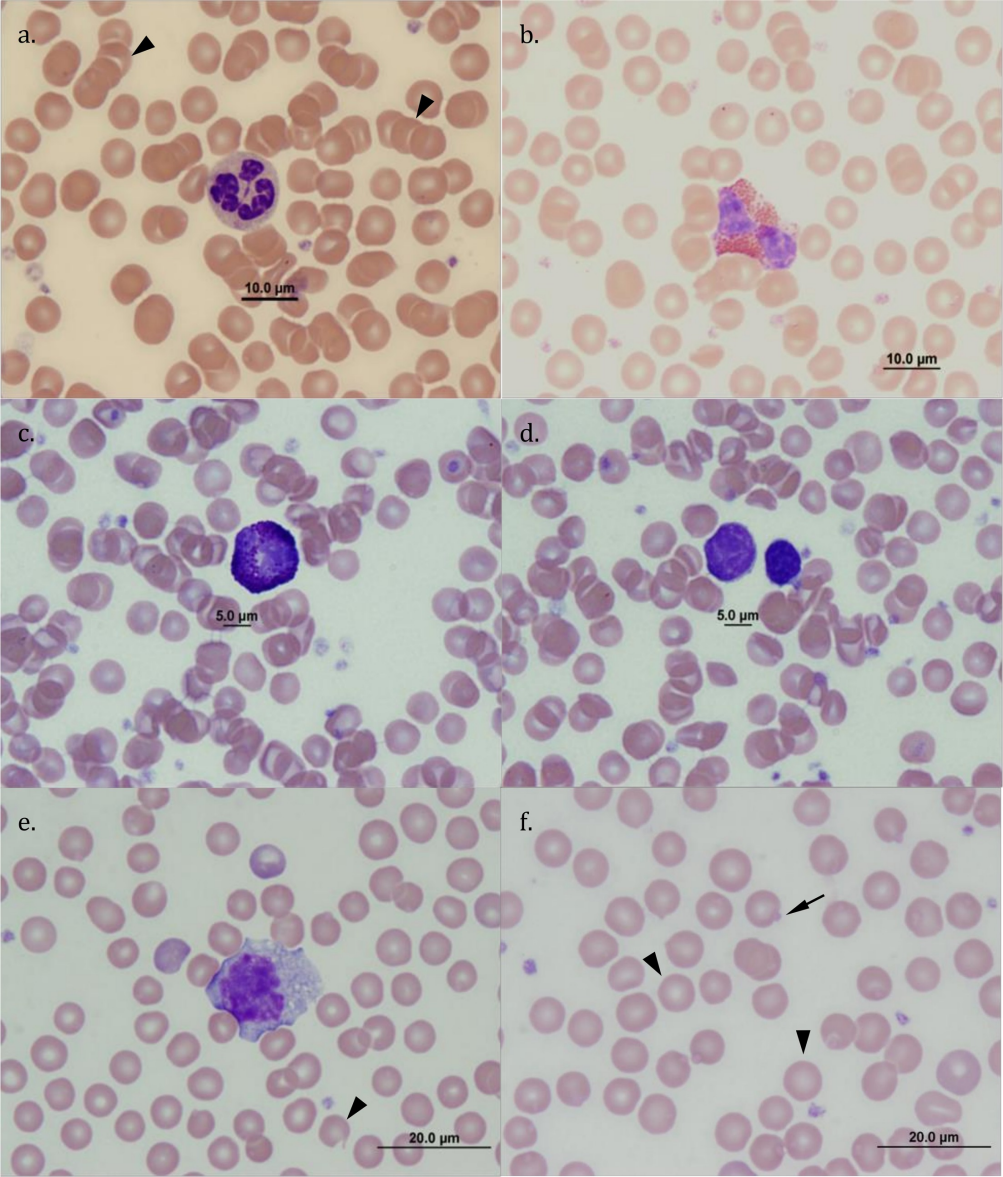
Photomicrographs of reference leukocytes and red blood cells observed in blood smears of quokkas on Rottnest Island and the mainland of Western Australia. (a) neutrophil, key features: polylobated nucleus with 3–6 lobes, coarsely clumped chromatin, primary cytoplasmic granules -azurophilic-; also present, atypical arrangement of erythrocytes: Rouleaux formation (arrowheads); (b) eosinophil, key features: polylobated nucleus with 2–3 lobes, coarsely clumped chromatin but less dense than neutrophils, prominent secondary eosinophilic cytoplasmic granules; (c) basophil, key features: intense secondary basophilic cytoplasmic granules; (d) lymphocytes, key features: darkly staining chromatin with no apparent nucleolus, cytoplasm presents as a “rim” and appears finely or coarsely granular, nuclear:cytoplasm (N:C) ratio smaller than that of other leukocytes; (e) monocyte, key features: indented to irregularly shaped nucleus, reticular chromatin, large amount of pale, grey to basophilic cytoplasm, often with vacuoles, overall size of the cell is greater than all other leukocytes; a keratocyte (i.e. poikilocyte; arrowhead) is also present; (f) erythrocytes with normal morphology (arrowheads), and with erythtocytic inclusions (e.g. Heinz body -arrow-). All photomicrographs: original magnification x1000-, staining Wright-Giemsa.

**Fig 2:**
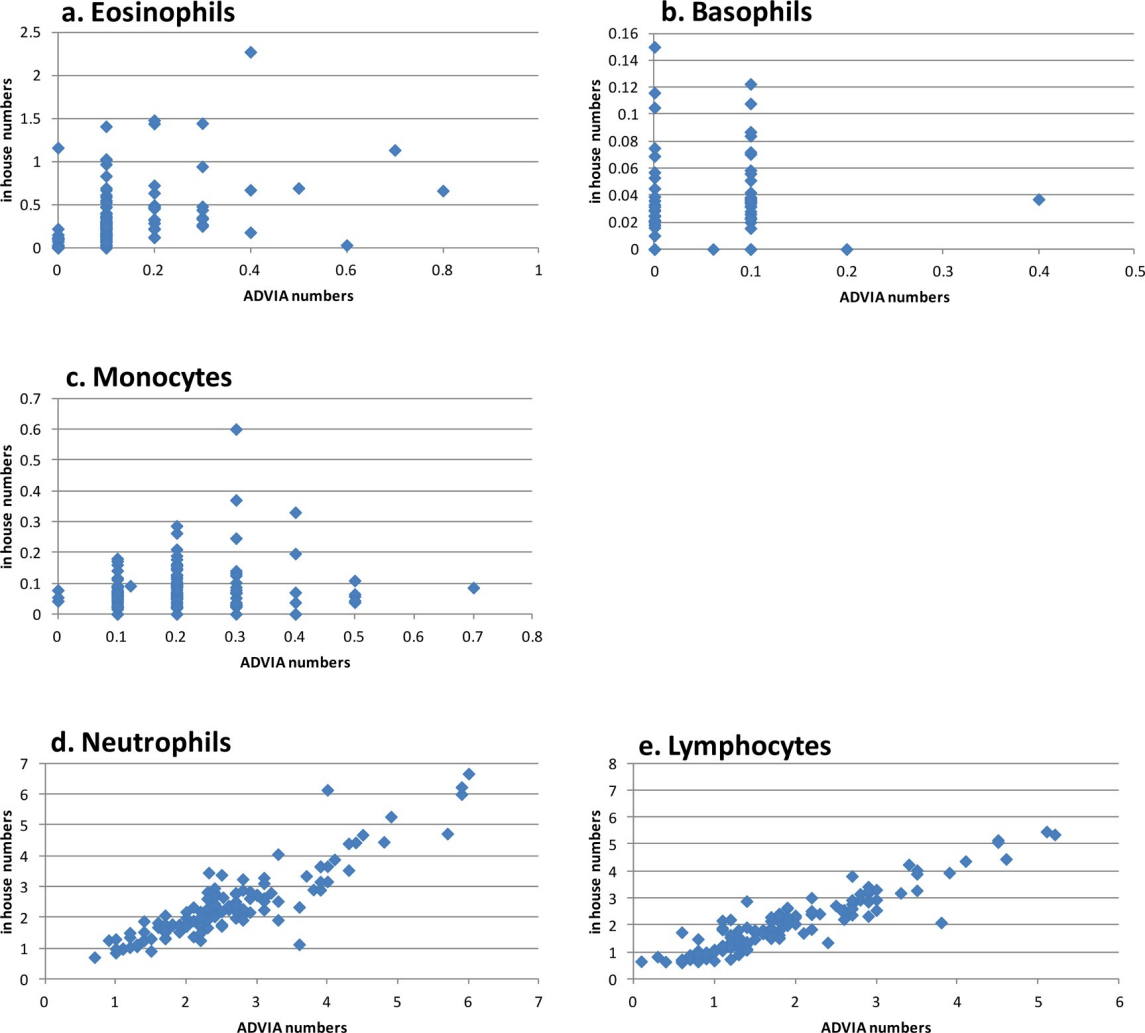
Correlation (r) of white blood cell classification by the ADVIA-120® with the 200-cell manual differential method. Eosinophils (a. r = 0.41), Basophils (b. r = 0.153), Monocytes (c. r = 0.04), Neutrophils (d. r = 0.90), and Lymphocytes (e. r = 0.93). Distances along the axes are unit-less, therefore the positions of the points in the plots are relative distances from one another rather than absolute differences read in these units.

Light scattering and impedance (i.e. volumetric sizing) standards do not exist for quokkas, and the algorithms of the ADVIA-120® cannot adjust to morphological variations of the cells of interest [26–28], and so differential leukocyte counts obtained through the ADVIA-120® automated haematology analyser were compared to manual (in house) cell counts. For the manual cell count, neutrophils (NEUT), eosinophils (EOS), basophils (BASO), lymphocytes (LYMPH) and monocytes (MONO) were identified based on published information for these cell populations in quokkas [29] (Fig 1). There was a low correlation (*r* ≤ 0.64) for EOS, BASO and MONO between the in house (200 cells) and the ADVIA-120® counts (Fig 2). By contrast, there was strong correlation (*r* = 0.85–0.94) between estimates for NEUT and LYMPH (Fig 2). The ADVIA® 120 data included fewer EOS and more NEUT than the manual differential (Fig 2). Inspection of the manual differential data revealed that the ADVIA® 120 included EOS within the NEUT count. The ADVIA-120® nucleated blood cell count was then corrected by subtracting the polychromatophilic erythrocyte count.

#### Blood chemistry (BLC)

BLC analytes (except vitamin E) (Table 1) were measured in an RX Daytona™ automatic biochemistry analyser (Randox Laboratories). For vitamin E, plasma samples were sent to the Animal Health Laboratories at Department of Agriculture and Food of Western Australia for vitamin E analyses using the method of McMurray and Blanchflower [30]. The chromatographic separation was performed with an Agilent HPLC system (1100) on a Zorbax SB-C18 column (3 mm x 150 mm, 3.5 μm) (Agilent Technologies) with a methanol mobile phase. Alpha-tocopherol (vitamin E) was quantified using fluorescence detection (ex. 296 nm and em. 330 nm).

#### Peripheral blood cell morphology (PBCM)

Blood smears (EDTA) were stained with a Hema-tek® Slide Stainer using Hema-tek® Wright’s Giemsa stain (Ames Company, Miles Laboratories). Smears were then assessed using light microscopy to determine the differential leukocyte count (200 leukocytes) and polychromatophilic erythrocyte count (1,000 erythrocytes) at x400 magnification. Leukocytes were identified according to previously published descriptions [29]. Additionally, erythrocyte morphology was assessed, including poikilocytes (i.e. atypically shaped erythrocytes) such as echinocytes, keratocytes and spherocytes; structures inside the erythrocyte (e.g. Heinz bodies and Howell-Jolly bodies), and atypical erythrocyte arrangement (e.g. Rouleaux formation) (Fig 1). Atypical leukocytes were also recorded (e.g. toxic changes in neutrophils: Döhle bodies), along with free-circulating parasitic organisms (i.e. microfilariae and trypanosomes). Abnormal cell morphologies, such as Döhle bodies and Heinz bodies, were assessed during the 200-leukocyte differential count at x1000 magnification. Presence and numbers of microfilariae were obtained by examining the complete blood smear at x40 magnification but anatomical characteristics of microfilariae were recorded at x400 or x1000 magnification. The presence of intraerythrocytic organisms was assessed by examining 100 fields within the monolayer and feathered regions of the blood smear combined; first at x400 magnification, and subsequently at x1000 magnification. ImageJ v1.49t [31] was used to make life measurements of microfilariae. The presence of piroplasms (*Babesia* and *Theileria*) and trypanosomes was determined by PCR as described above.

### Statistical analyses

Analyses were calculated on different sample sizes as in some instances there was an insufficient volume of blood, there was haemolysis, and/or blood smears did not have adequate cell distribution and quality. Sample sizes are indicated in the tables where individual datasets are presented.

#### Site, sample time and sex effects

HMT, BLC and PBCM datasets were explored with non-metric Multidimensional Scaling (nMDS) using a Bray-Curtis similarity measure [32] (PAST v. 3.02 [33]). nMDS allows simultaneous analysis of multiple measures (non-independent of each other) to create a profile that represents the similarity of the suite of measures which can then be statistically analysed using an Analysis of Similarity (ANOSIM) [34]. Analyses were performed on HMT and BLC data sets separately due to differences in sample sizes between these datasets. Data for HCT and BLC measures were range-standardised to a scale between 0 and 1; binary data for PBCM measures did not require range standardisation. Two- or three-dimensional models were selected according to the model that had the lowest stress statistic.

A two-way ANOSIM (PAST) with *sex* and *site* as independent factors was used to analyse patterns in HMT and BLC datasets for all samples; Rottnest Island samples were subsequently re-analysed with *sample time* and *sex* as independent factors. To establish the contribution of each dependant variable to the overall similarity or dissimilarity observed between groups, a pairwise similarity percentage (SIMPER) (PAST) [34] analysis using the Bray-Curtis similarity measure [32] was carried out.

#### Comparison of vitamin E for captive and wild-caught quokkas

A Mann-Whitney U test (STATISTICA v. 9.1, StatSoft Inc.) was used to explore significant differences between plasma concentrations of vitamin E from captive quokkas (Perth Zoo; n = 8) that were originally sourced from Rottnest Island, and plasma concentrations of vitamin E of free-ranging animals from Rottnest Island at the same time of year (i.e. winter, n = 29).

#### Associations with potential infectious agents (IA)

To determine the effect of IA presence on blood analyte profiles, nMDS (with Bray-Curtis similarity) were performed to holistically quantify the blood profiles; HMT and BLC datasets were handled separately due to different sample sizes for animals with complete HMT, BLC and IA data. To identify which factors most strongly determined the nMDS scores for each dimension, Spearman Rank Order correlation matrix (STATISTICA) was carried out comparing the nMDS axis scores and analyte values. Second, multiple regression (STATISTICA) was then used to determine the correlations (*R* and *F* statistics) and strength (*Beta* coefficient) of the relationships between each of the nMDS axes and the presence/absence of each IA. To account for site differences, *site* was also included as an independent factor in this analysis. Due to the low frequency of detection, *Babesia* spp. and trypanosomes were excluded from both HMT and BLC datasets, while *Cryptococcus* spp. was excluded from the BLC dataset analysis. *Cryptococcus* spp. and saprophytic fungi were assessed separately in the haematology dataset as more than one positive in each group was available for analysis.

To identify common blood analyte profiles, unsupervised hierarchical cluster analyses (PAST) using an unweighted pair-group average algorithm and a Euclidean similarity index [35] were run for the HMT and BLC datasets separately. Each cluster analysis is presented in the form of a dendrogram with bootstrapping analysis with 100 replications. Dendrograms were then coupled with matrix plots (PAST) of the HMT, BLC and nMDS axis data.

Separate Mann-Whitney U tests were used to determine significant differences in WBC and LYMPH between Flower cell-positive and Flower cell-negative animals on Rottnest Island. Chi-square was also used to determine significance in the variation of Flower cells (only observed in Rottnest Island peripheral blood smears) across sex and sample times in the Rottnest Island sample.

#### Reference intervals

Criteria for the inclusion of reference individuals were a cloacal temperature between 36.5°C and 38.5°C [36] taken immediately after anaesthetic induction, no evidence of dehydration, a body condition score between 2 and 4 (inclusive) out of 5, mucous membranes that were pink or pale pink and moist, and the absence of other obvious signs of disease (e.g. dermatitis, purulent nasal discharge). Blood sample quality was recorded at the times of collection and analysis. Samples were excluded if haemolysis and/or lipaemia were noted.

Reference intervals for selected HMT and BLC analytes were explored and constructed using Reference Value Advisor v.2.1 [37], which has been used for a number of other animal species [38–40] and is accepted by the American Society for Veterinary Clinical Pathology as a tool that adheres to the most recent guidelines (EP28-A3C) of the International Federation of Clinical Chemistry (IFCC) and the Clinical and Laboratory Standards Institute (CLSI) [41]. A test for normality (Anderson-Darling) was performed for all analytes, and all data were transformed using the generalized Box-Cox transformation. Outliers were detected with Dixon-Reed and Tukey’s tests.

As recommended by the revised CLSI guidelines, the non-parametric calculated reference interval is reported when data of a sufficient sample size (i.e. ≥ 40, ideally ≥ 120) [41] has a unimodal distribution [37]. Confidence intervals for the non-parametric calculated reference interval were computed using a bootstrap method [37]. This approach was used to construct HMT and BLC reference intervals for the Rottnest Island subpopulation. Reference intervals for the mainland dataset (n< 40), were those given by the robust method with Box-Cox transformed data when possible; otherwise reference intervals are given using the robust method with untransformed data. In this case, confidence intervals were computed using a non-parametric bootstrap method [37].

Manual differential leukocyte count data were used when calculating the corresponding reference intervals. We calculated the correlation coefficients (*r*) using least squares linear regression analysis to compare the results of the ADVIA-120® for NEUT, LYMPH, EOS, BASO and MONO, to those obtained through the manual differential count (the manual counts were used for all other analyses). For the Rottnest Island sample, the reference interval for platelet concentration was constructed after removing samples that contained platelet clumps on the blood smear. Comparison of HMT and BLC values between mainland locations was not done as the model was too uneven.

#### General

Odds ratio (OR) and its corresponding 95% CI when presented, were calculated using Woolf’s method [42]. All other 95% CI for estimates of proportions (i.e. prevalence), were calculated using the Wilson model for n ≤ 40, and the Jeffreys model for n > 40 [25]. For all analyses, statistical significance was set to *p* < 0.05.

## Results

Blood samples were collected from 150 quokkas.

### Site, sample time and sex effects

#### Combined data sample (Rottnest Island-mainland)

**Fig 3:**
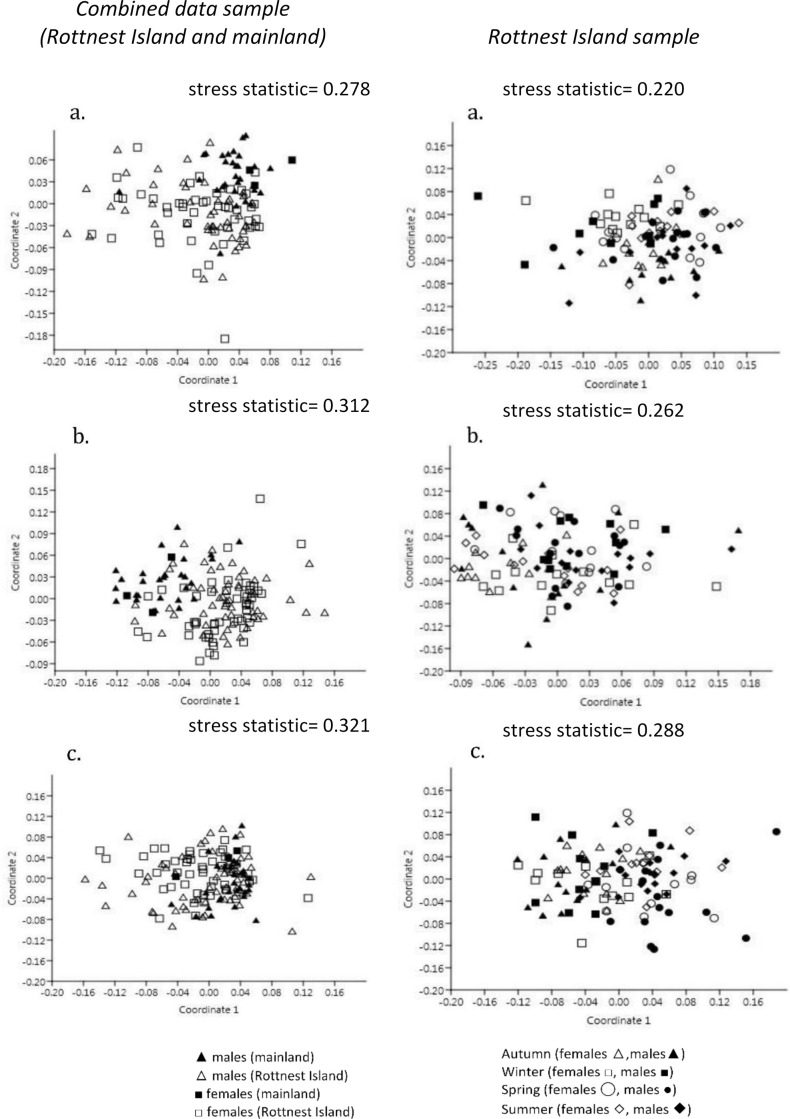
Non-metric MDS plots illustrating the structural dissimilarity in (a) haematology HMT (RI n = 96, ML n = 32), (b) blood chemistry BLC (RI n = 106, ML n = 32), and (c) peripheral blood cell morphology PBCM (RI n = 107, ML n = 34) for quokkas from Rottnest Island (open symbols) and the mainland (filled symbols) (left hand panel) or quokkas on Rottnest Island sampled at four times of the year (‘seasons’: autumn: triangles, winter: squares, spring: circles, and summer: diamonds) (right-hand panel). Key legend applies for all plots. Note that the distances along the axes are unit-less, therefore the positions of the points in the plots are relative distances from one another rather than absolute differences read in these units.

There were significant site differences (i.e. Rottnest Island v mainland) in HMT data (ANOSIM, *R* = 0.296, *p* = 0.003; Table 2), with evident clustering of HMT data by site on the nMDS plot (Fig 3). *Site* differences in HMT were most evident for RBC and LYMPH (SIMPER contribution percentage, Ct%, > 10%; Table 1A). Mainland animals had higher mean RBC, PCV, HGB, CHCM, NEUT and BASO, while Rottnest Island animals had greater LYMPH, EOS, WBC, and MCV. Significant *site* differences in BLC (ANOSIM, *R* = 0.463, *p* = 0.001; Table 2) were evident on the nMDS plot (Fig 3). The most marked difference in BLC was the 8-fold greater CK (Ct% > 10%; Table 1B) for mainland quokkas compared to Rottnest Island animals. Significant *site* differences in PBCM (ANOSIM, *R* = 0.434, *p* = 0.001; Table 2) were most evident in the incidence of Heinz bodies, acanthocytes, Rouleaux formation, and hypochromasia (Ct% > 10%; Table 1C).

**Table 2:** Summary of two-way ANOSIM (*R* statistic and p value) of blood analytes (see footnote) for Rottnest Island (RI) and mainland (ML) (left hand column) and Rottnest Island only (right hand column) quokkas.

		Rottnest Island v Mainland			Rottnest Island (four timepoints)		
	Variables	Factor	R	*p*	Factor	R	*p*
a.	HMT (RI n = 96; ML n = 32)	Site	0.296	**0.003**	Sample time	0.183	**0.001**
		Sex	0.045	0.267	Sex	0.039	0.084
b.	BLC (RI n = 106; ML n = 32)	Site	0.463	**0.001**	Sample time	0.160	**0.001**
		Sex	-0.052	0.681	Sex	0.023	0.190
c.	PBCM (RI n = 107; ML n = 34)	Site	0.434	**0.001**	Sample time	0.304	**0.001**
		Sex	-0.043	0.522	Sex	0.035	0.114

(a) haematology (HMT) analytes from whole blood in EDTA: corrected WBC, RBC, HGB, PCV, and absolute counts for leukocytes obtained with a manual differential on a blood smear.

(b) blood chemistry (BLC) analytes from whole blood in Lithium-Heparin: ALP, ALT, AST, CK, PROT, ALB, GLOB, CALC, PHOSP, CHOL, BILT, GLUC, CREAT, UREA and Vitamin E.

(c) peripheral blood cell morphology (PBCM)

There were no *sex* differences in the HMT, BLC or PBCM data (ANOSIM; *p* > 0.05; Table 2) and no clustering by *sex* evident for the nMDS plots (Fig 3).

#### Rottnest Island sample

**Fig 4:**
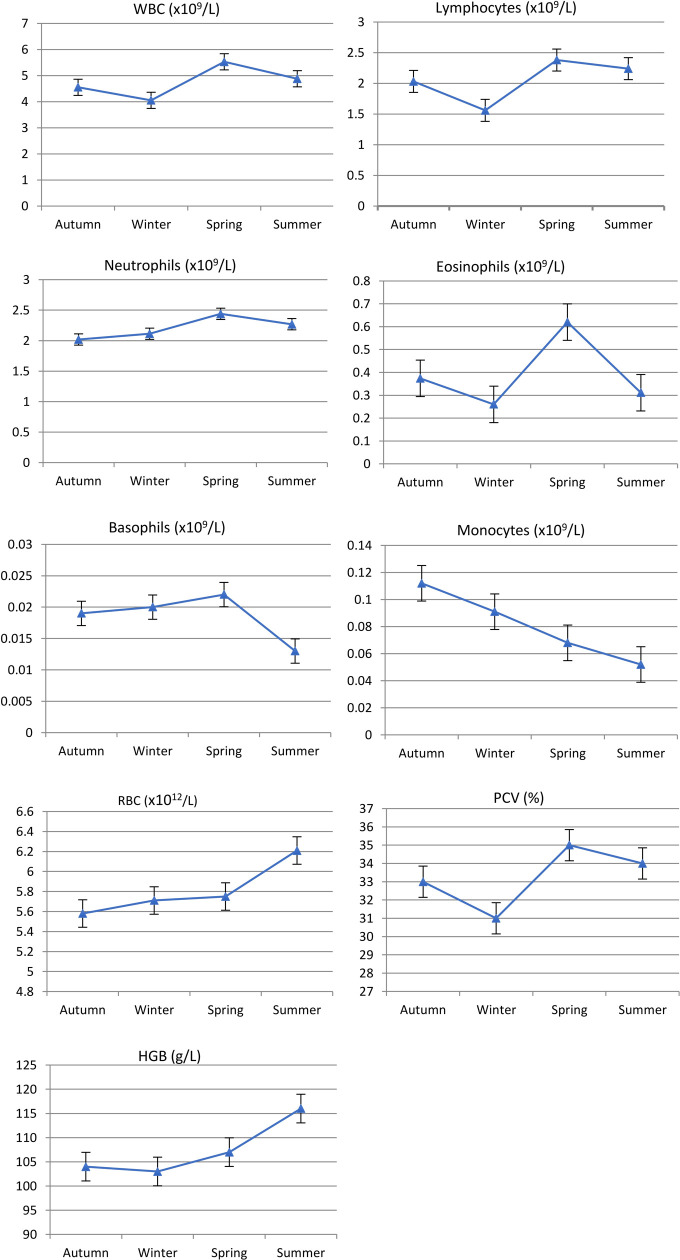
Linear plots for haematocrit (HMT) analytes across seasons for quokkas trapped on Rottnest Island between March and December 2011. Autumn n = 20, Winter n = 21, Spring n = 27, Summer n = 28.

**Fig 5:**
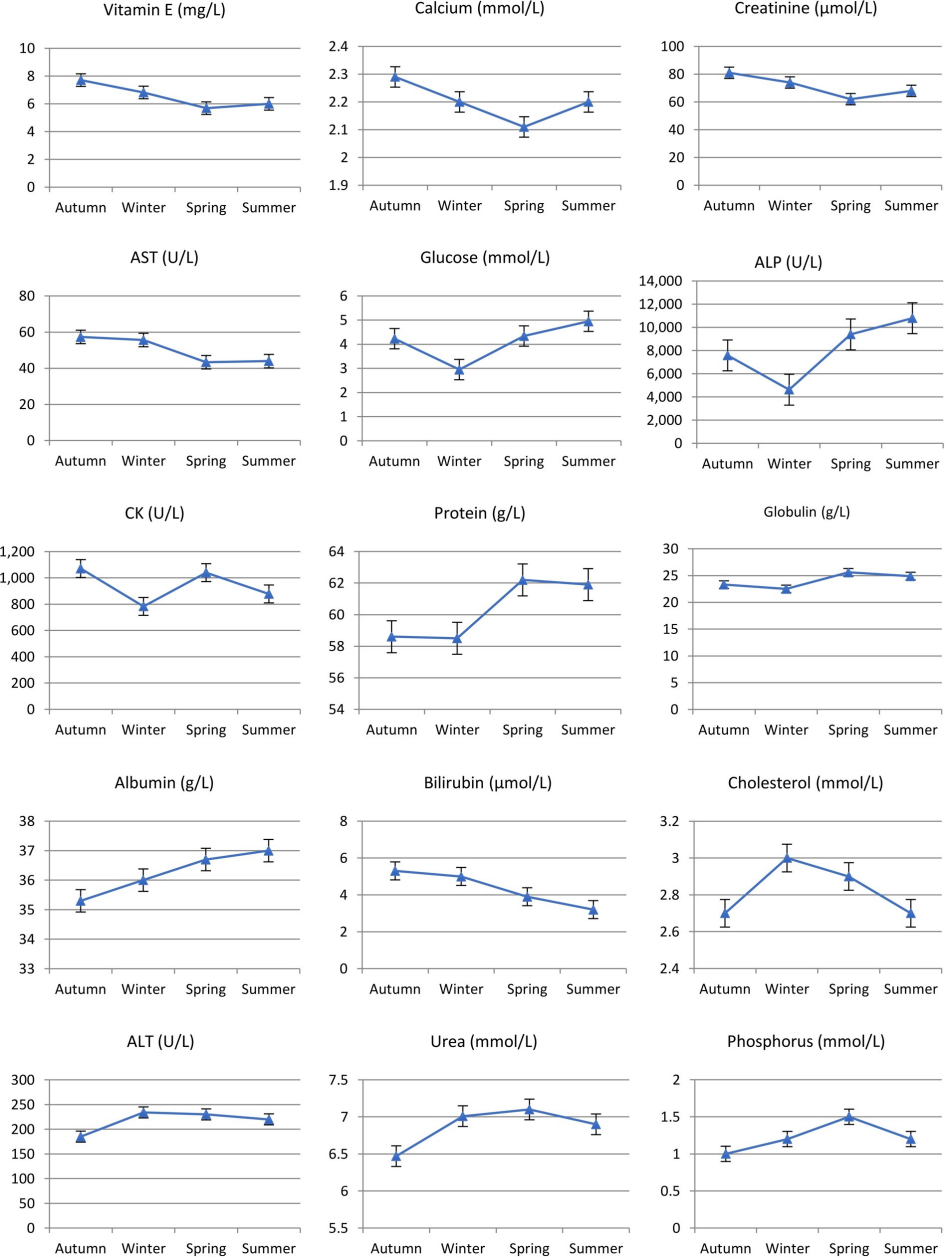
Linear plots for blood chemistry (BLC) analytes across seasons for quokkas trapped on Rottnest Island between March and December 2011. Autumn n = 26, Winter n = 25, Spring n = 26, Summer n = 29.

**Fig 6:**
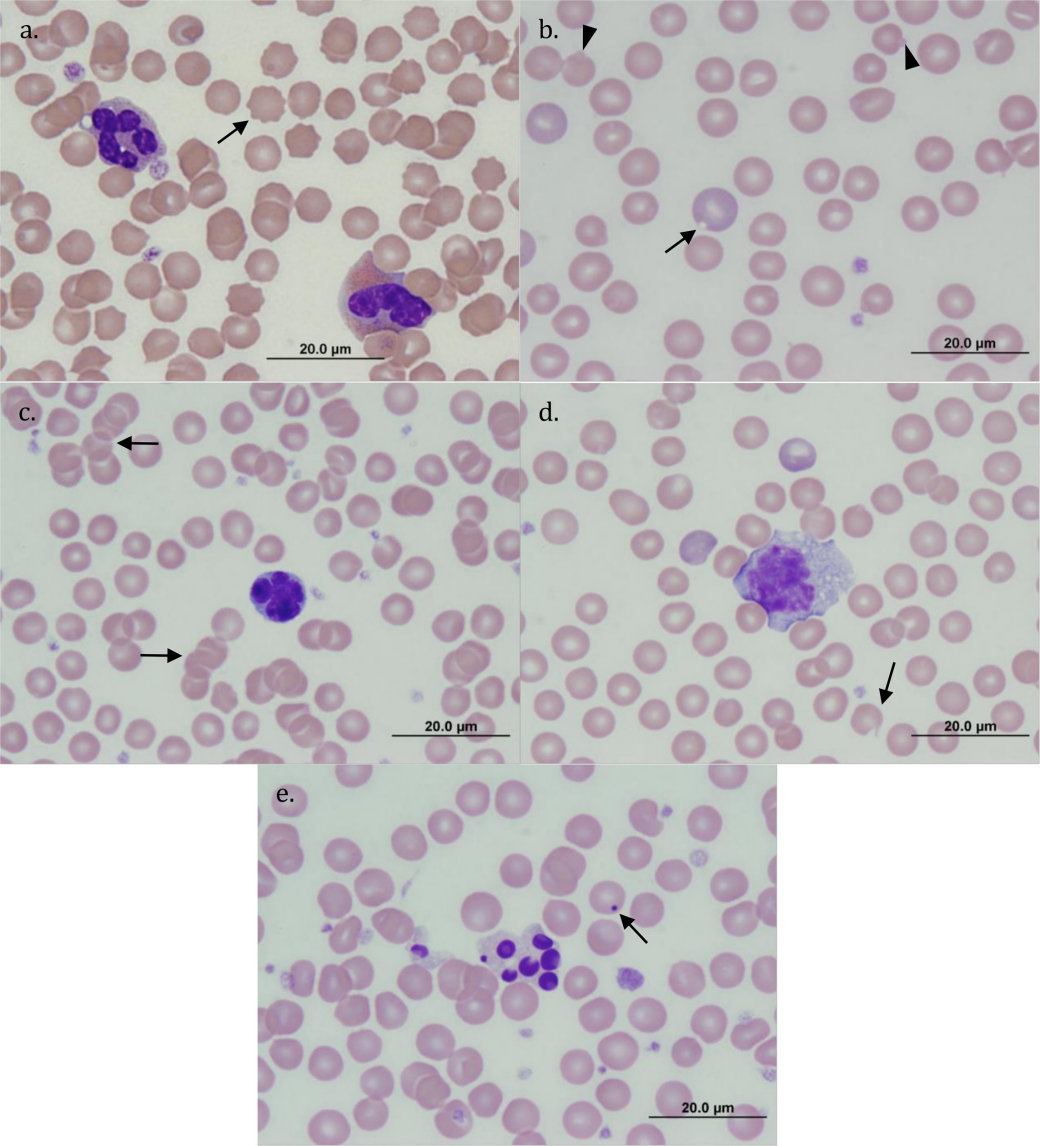
Some erythrocytic morphologies and intraerythrocytic inclusions found in peripheral blood smears of quokkas on Rottnest Island (also found in quokkas on mainland Western Australia). (a) echinocytes (arrow), two large cells present: neutrophil (left), eosinophil (right); (b) Heinz bodies (arrowheads), blister polychromatophilic cell (arrow), anisocytosis is also present in this plate; (c) Rouleaux formation (arrowheads), lymphocyte with a flower-like nucleus (large cell centre); (d) keratocyte (arrow), monocyte (centre) and polychromatophilic erythrocytes present; (e) Howell-Jolly body (arrow), leukocyte with fragmented nucleus (large cell centre). All images: magnification x1000, staining Wright-Giemsa.

There were significant sample time differences in HMT (ANOSIM, R = 0.183, *p* = 0.001; Table 2) for Rottnest Island animals although clustering was not strongly evident in the nMDS plot (Fig 3). Differences in HMT with sampling time were most pronounced for (in order of greatest effect to least effect with Ct% > 10%; Table 3A and Fig 4) MONO (being highest in autumn and lowest in summer), LYMPH and WBC (both highest in spring and lowest in winter) and MCV. Significant *sample time* differences in BLC (ANOSIM, *R* = 0.160, *p* = 0.001; Table 2 and Fig 5) were most evident (Ct% > 8%; Table 3B) in Vit. E (highest in autumn and lowest in spring/summer), CK (highest in autumn and lowest in winter), TP (highest in spring/summer and lowest in autumn/winter) and GLUC (highest in summer and lowest in winter). Significant *sample time* differences in PBCM (ANOSIM, *R* = 0.304, *p* = 0.001; Table 2) were most evident in the incidence of Heinz bodies, acanthocytes and Rouleaux formation (Ct% > 11; Table 3C). Photomicrographs of some red blood cell morphologies are presented in Fig 6.

**Table 3:** SIMPER analysis indicating the percent contribution (Ct %) of specific variables to the observed time of year differences in (a) Haematology (HMT), (b) Blood chemistry (BLC), and (c) Peripheral blood cell morphology (PBCM) profiles of quokkas from Rottnest Island. For (a and b), mean and standard deviation. For (c), the number of animals (percentage of sampled animals) with 95% confidence intervals(§). Abbreviations and units as per Table 1.

		Autumn		Winter		Spring		Summer	
Analyte	Ct %	$\bar{x}$	SD	$\bar{x}$	SD	$\bar{x}$	SD	$\bar{x}$	SD
a) Haematology (HMT) (24.62*) autumn n = 20, winter n = 21, spring n = 27, summer n = 28									
MONO	12	0.112	0.112	0.091	0.064	0.068	0.068	0.052	0.045
LYMPH	12	2.032	0.84	1.56	0.75	2.38	1.29	2.24	1.16
WBC	11	4.55	1.25	4.052	1.201	5.53	1.75	4.88	1.62
MCV	11	57.6	2.59	65.2	2.96	60.4	2.59	60.9	2.56
BASO	9.7	0.019	0.035	0.02	0.022	0.022	0.031	0.013	0.026
RBC	8.8	5.58	0.61	5.71	0.97	5.75	0.74	6.21	0.98
EOS	8.3	0.374	0.321	0.26	0.188	0.62	0.509	0.311	0.218
NEUT	8.1	2.019	0.53	2.113	0.606	2.44	1.16	2.27	1.036
PCV	7.6	33	3.8	31	3.8	35	4.5	34	4.7
HGB	6.6	104	15	103	17	107	12	116	15
CHCM	5.8	323	22	294	6.8	306	11	306	5.9
b) Blood chemistry (BLC) (19.72*) autumn n = 26, winter n = 25, spring n = 26, summer n = 29									
Vit. E	9.1	7.71	1.86	6.82	1.77	5.69	1.65	6	1.27
CK	9	1,071	1,249	783	589	1,040	1,040	878	1,984
TP	8.2	58.6	4.15	58.5	3.59	62.2	5.25	61.9	4.15
GLUC	8.1	4.23	1.79	2.95	2.03	4.34	1.96	4.95	2.57
CHOL	7.8	2.7	0.6	3	0.5	2.9	0.5	2.7	0.5
PHOS	7.4	1	0.4	1.2	0.4	1.5	0.5	1.2	0.5
GLOB	7.1	23.3	2.98	22.5	3.14	25.6	4.35	24.9	3.71
ALT	6.9	185	37	234	74.7	230	56.3	220	65.3
CALC	6.5	2.29	0.197	2.2	0.172	2.11	0.162	2.2	0.23
BILT	6.1	5.3	1.8	5	1.6	3.9	0.6	3.2	1.4
ALB	5.5	35.3	2.15	36	1.36	36.7	2.01	37	1.63
ALP	5.4	7,582	11,043	4,622	2,361	9,390	10,168	10,787	12,492
AST	4.9	57.3	30.7	55.6	48.3	43.3	15.7	43.9	22.9
CREAT	4.2	81	20	74	18	62	9.1	68	8.2
UREA	3.8	6.47	1.88	7.01	1.53	7.1	1.54	6.9	1.16
c) Peripheral blood cell morphology (PBCM) (25.5*) autumn n = 28, winter n = 23, spring n = 27, summer n = 28									
		n (%)	CI	n (%)	CI	n (%)	CI	n (%)	CI
Heinz Bodies	12	10 (36%)	21–54	17 (74%)	53–87	9 (33%)	19–52	22 (79%)	60–90
Acanthocytes	12	11 (39%)	24–58	15 (65%)	45–81	8 (30%)	16–48	18 (64%)	46–79
Rouleaux Formation	12	13 (46%)	29–64	15 (65%)	45–81	11 (41%)	24–59	7 (25%)	13–43
Echinocytes	9.2	11 (39%)	24–58	5 (22%)	9.0–42	6 (22%)	11–41	8 (29%)	15–47
Schistocytes	8.4	13 (46%)	29–64	3 (13%)	4.0–32	7 (26%)	13–45	3 (11%)	4.0–27
Flower Cells	8.3	5 (18%)	8.0–36	9 (39%)	22–59	12 (44%)	28–63	2 (7.1%)	2.0–23
Keratocytes	8.1	11 (39%)	24–58	9 (39%)	22–0.59	1 (3.7%)	1.0–18	3 (11%)	4.0–27
Anisocytosis	7.9	24 (86%)	68–94	23 (100%)	86–100	19 (70%)	51–84	20 (71%)	53–84
Poikilocytosis	5.8	26 (93%)	77–98	21 (91%)	73–98	19 (70%)	51–84	28 (100%)	88–100
Hypochromasia	4.6	24 (86%)	68–94	22 (96%)	79–99	22 (81%)	63–92	28 (100%)	88–100
nRBCs	4.4	28 (100%)	88–100	17 (74%)	53–87	24 (89%)	72–96	28 (100%)	88–100
Polychromasia	4.1	26 (93%)	77–98	21 (91%)	73–98	27 (100%)	87–100	24 (86%)	68–94
Howell-Jolly Bodies	3.4	27 (96%)	82–99	21 (91%)	73–98	25 (93%)	77–98	26 (93%)	77–98
Reactive Lymphocytes	0.6	28 (100%)	88–100	23 (100%)	86–100	26 (96%)	82–99	28 (100%)	88–100

**§** calculated using the Wilson model [25].

There were no *sex* differences in the HMT, BLC or PBCM data (ANOSIM; *p* > 0.05; Table 2) and no clustering by *sex* evident for the nMDS plots (Fig 3).

#### Comparison of vitamin E for captive and wild-caught quokkas.

**Fig 7:**
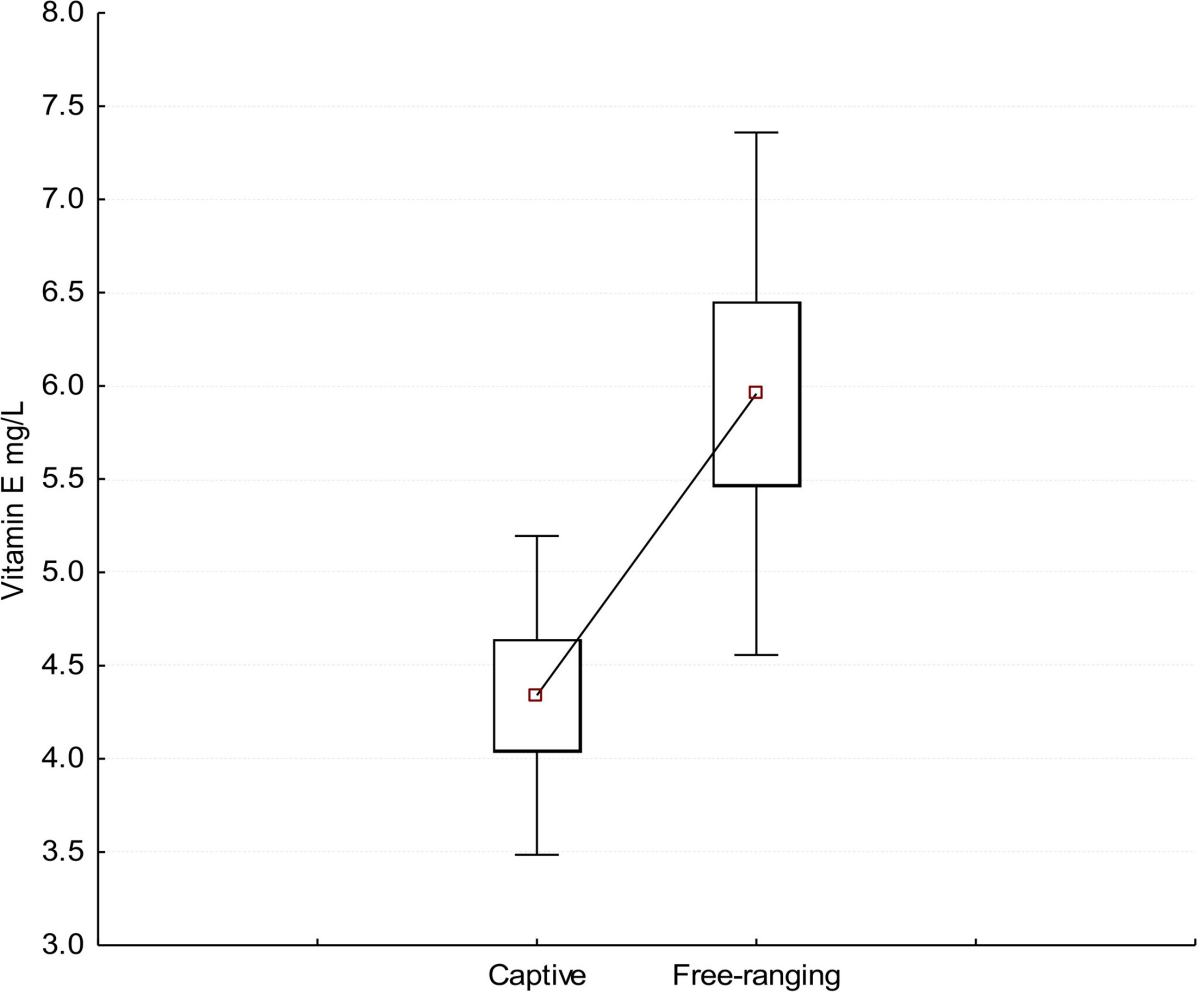
Box-plot of the concentration of vitamin E (mg/L) in plasma in winter for quokkas from Rottnest Island (free-ranging, n = 29) and quokkas from Perth Zoo (captive, n = 8).

Captive individuals had significantly lower mean plasma vitamin E levels (mean ± SD = 4.34 ± 0.86 mg/L; median = 4.15 mg/L) than free-ranging quokkas on Rottnest Island (mean ± SD = 6.00 ± 1.27 mg/L; median = 5.75 mg/L) (U_n = 37_ = 34.5, *p* = 0.003) (Fig 7).

### Atypical cells

**Fig 8:**
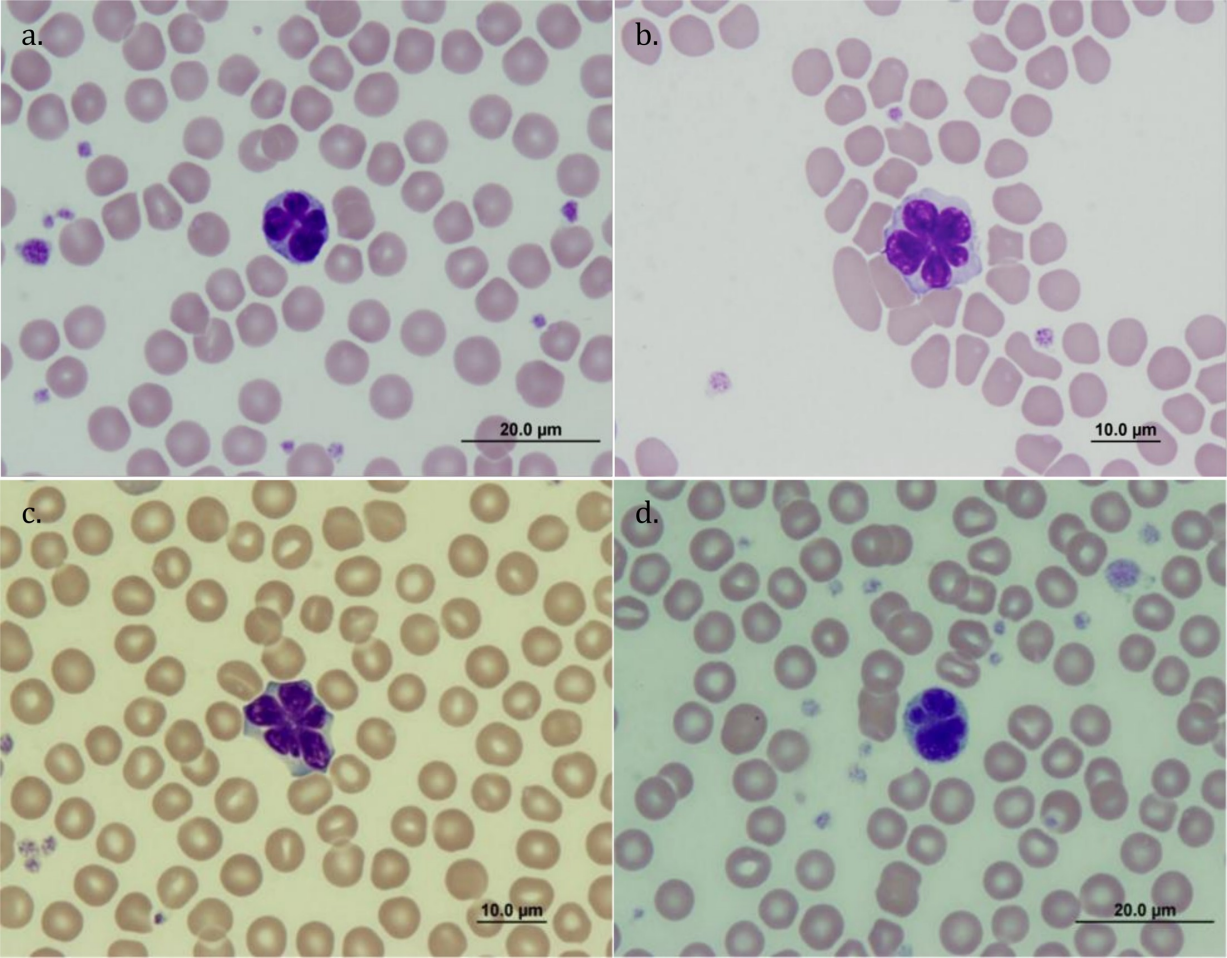
Microphotographs of atypical lymphocytes observed in peripheral blood smears of quokkas on Rottnest Island (a-c) atypical lymphocytes exhibiting a “flower” shape-like polylobated nucleus with condensed homogeneous chromatin; (d) atypical lymphocyte exhibiting a polylobated nucleus resembling “prototype lymphocytes”; plates a-d: original magnification x1000, stain: Wright-Giemsa.

Leukocytes with polylobated nuclei and condensed and homogeneous chromatin, which were presumed to be Flower cells (Fig 8A–8C), as well as leukocytes with polylobated nuclei but lobes arranged in no specific shape (Fig 8C), were observed in Rottnest Island quokkas. The proportion of animals where Flower cells were detected was 21% (23/108, 95% CI 14.4–29.7%), with 1–6 cells per 200 leukocytes. Neither the sex of the quokka (*χ²*_1_ = 0.02, *p* = 0.892) nor the time of year ('*sample time*') that the blood was collected (*χ²*_3_ = 0.86, *p* = 0.354) was associated with their presence. There were, however, significant differences in the average WBC between Flower cell-positive (5.30 x 10^9^/L) and Flower cell-negative (4.45 x 10^9^/L) animals (U_n = 108_ = 668, *p* = 0.026), but not in the LYMPH count (U_n = 108_ = 806, *p* = 0.201). By contrast, Flower cells were not observed in the peripheral blood of mainland animals.

### Associations with infectious agents (IA)

Infectious agents were detected in 24 out of 58 quokkas (41.4%, 95% CI 29.4–54.2%); Table 4). In all 24 cases, more than one IA was detected. The most common IA were *Salmonella* (present in 12 Rottnest Island animals), MaHV-6 (present in 11 mainland animals), *Theileria* spp. (present in 10 mainland animals) and microfilariae (present in 11 animals; both locations). Of the mixed infections detected, the most prevalent were *Salmonella*–microfilariae (eight Rottnest Island animals) and MaHV-6–*Theileria* spp. (10 mainland animals).

**Table 4:** Summary of mixed infections captured in both haematology and blood chemistry datasets combined, for Rottnest Island and mainland animals that tested positive (+) for eight infectious agents.

Site	*Salmonella*	Microfilariae	MaHV-6	*Theileria* spp.	*Cryptococcus* spp.	Saprophyte fungi	*Babesia* spp.	Trypanosomes	No. of animals	% of total	95% CI †
Rottnest Island n = 45	+						+		1	4.3	0.8–21.0
	+				+				1	4.3	0.8–21.0
				+	+				1	4.3	0.8–21.0
	+		+			+			2	3.60	0.7–11.0
	+	+			+				2	3.60	0.7–11.0
	+	+		+					2	3.60	0.7–11.0
	+	+							4	7.10	2.5–16.1
No. of animals with mixed infection on Rottnest Island									13	22.4	13.2–34.3
Mainland n = 13		+	+	+		+			1	4.3	0.8–21.0
		+	+	+					2	3.60	0.7–11.0
			+	+				+	1	4.3	0.8–21.0
			+	+					4	5.40	1.5–13.6
			+	+		+			2	3.60	0.7–11.0
			+			+			1	4.3	0.8–21.0
No. of animals with concomitant infection on the mainland									11	19	10.5–30.4
Total no. of animals with concomitant infection									24	41.4	29.4–54.2
Total no. of animals tested									58		

† 95% confidence intervals (CI) calculated using Woolf’s method [42].

When *site* differences were also taken into account in multiple regression analyses, there were additional significant effects of the presence of IA on the HMT and BLC profiles (Table 5). Most markedly, the presence of *Salmonella* was significantly associated with values on all three nMDS dimensions for HMT, specifically with high EOS, BASO and CHCM values. The presence of *Salmonella* was also significantly associated with high ALT and AST values for the BLC profile. The presence of microfilariae was associated with increased MONO, BILT and CHOL. The presence of MaHV-6 was strongly associated with increased RBC, HGB, PCV and WBC, as well as GLUC and UREA. The presence of *Theileria* spp. was strongly associated with increased RBC, HGB, MONO, and EOS, as well as PROT, ALB and PHOS. The presence of *Theileria* spp. was strongly associated with increased RBC, HGB, MONO, and EOS, as well as PROT, ALB and PHOS. The presence of saprophytic fungi was strongly associated with increased WBC, as well as GLUC and UREA. There were no changes in HMT associated with the presence of *Cryptococcus* spp. (Table 5); changes in the BLC profile was not tested for this IA.

**Table 5:** Correlations between three axes of a 3D non-metric Multidimensional Scaling (nMDS) model for (a) haematology (HMT) and (b) blood chemistry (BLC) analytes for Rottnest Island and mainland animals (combined sample) that were tested for eight infectious agents (IA). In this case, the three-dimensional model had less stress than the two-dimensional model. Values above the horizontal line for HMT and BLC are Spearman rank order correlation coefficients with the nMDS axes scores. Below the horizontal lines are results of multiple regression testing for relationships with *site* and the presence/absence of six (HMT) or five (BLC) of these IA. Colour shading indicates high to low correlations of HMT (red to blue) and BLC (red to green).

	Analyte	nMDS axis 1		nMDS axis 2		nMDS axis 3	
a) HMT	WBC	0.646		0.206		-0.032	
n = 56	NEUT	0.469		0.177		0.006	
	EOS	-0.198		0.061		0.195	
	BASO	0.253		0.121		-0.289	
	LYMPH	0.249		0.067		0.064	
	MONO	0.403		-0.320		-0.240	
	RBC	0.838		0.108		-0.150	
	HGB	0.831		0.071		-0.191	
	PCV	0.670		-0.031		-0.071	
	CHCM	0.341		0.104		-0.262	
		*R* = 0.912; *F*_7,48_ = 33.9		*R* = 0.926; *F*_7,48_ = 41.4		*R* = 0.916; *F*_7,48_ = 35.8	
		Beta	*p*	Beta	*p*	Beta	*p*
	Site	**0.293**	**0.015**	-0.160	0.139	**-0.364**	**0.002**
	*Salmonella*	**-0.327**	**0.001**	**-0.136**	**0.035**	**-0.780**	**0.001**
	Microfilariae	**0.136**	**0.033**	**-0.768**	**0.001**	**-0.176**	**0.006**
	MaHV-6	0.221	0.077	0.188	0.101	**-0.262**	**0.034**
	*Theileria* spp.	**0.274**	**0.002**	**-0.233**	**0.003**	**0.231**	**0.006**
	*Cryptococcus* spp.	0.050	0.428	-0.057	0.320	-0.028	0.642
	Saprophytic fungi	0.148	0.064	**0.220**	**0.004**	**-0.207**	**0.010**
b) BLC	ALP	-0.165		-0.010		0.269	
n = 46	ALT	0.516		-0.341		0.155	
	AST	0.482		-0.328		0.216	
	CK	0.556		-0.245		0.219	
	GGT	0.043		-0.239		0.045	
	PROT	0.775		-0.128		0.124	
	ALB	0.780		0.033		0.107	
	GLOB	0.598		-0.162		0.107	
	CALC	0.408		-0.244		-0.112	
	PHOS	0.146		0.434		0.010	
	BILT	0.191		-0.011		-0.243	
	GLUC	0.203		-0.153		0.339	
	CHOL	0.272		0.320		-0.262	
	CREAT	0.383		-0.124		-0.081	
	UREA	0.332		-0.124		0.352	
	VIT. E	0.610		-0.075		-0.186	
		*R* = 0.911; *F*_6,39_ = 31.7		*R* = 0.923; *F*_6,39_ = 37.3		*R* = 0.824; *F*_6,39_ = 13.7	
		Beta	*p*	Beta	*p*	Beta	*p*
	Site	**0.521**	**0.001**	**-0.317**	**0.006**	-0.081	0.613
	*Salmonella* spp.	-0.030	0.683	**-0.740**	**<0.001**	-0.093	0.360
	Microfilariae	**0.207**	**0.005**	**-0.231**	**0.001**	**-0.619**	**0.001**
	MaHV-6	0.029	0.824	-0.128	0.290	0.248	0.164
	*Theileria* spp.	**0.322**	**0.001**	**0.460**	**0.001**	-0.143	0.241
	Saprophytic fungi	**0.250**	**0.011**	-0.150	0.092	**0.279**	**0.036**

**Fig 9:**
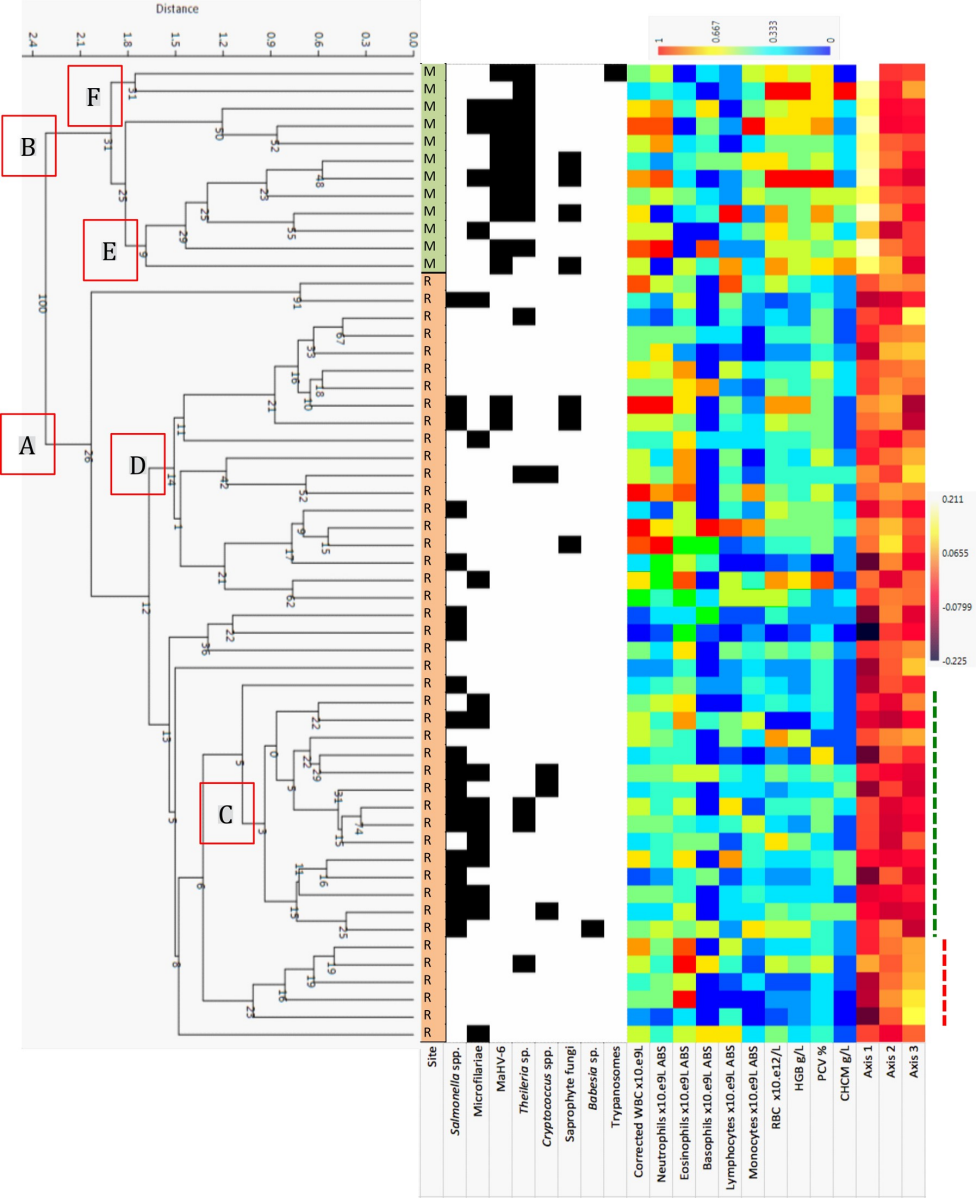
Cluster analysis and patterns of 10 HMT analytes and the three nMDS axis scores derived from these values for 12 animals from the mainland (M) and 44 animals from Rottnest Island (R) (each row represents an individual). Animals that tested positive for one of eight Infectious Agents (*Salmonella* spp., microfilariae, MaHV-6, *Theileria* spp., *Cryptococcus* spp., saprophyte fungi, *Babesia* spp., and trypanosomes) are shown as a black square. Clusters A (Rottnest Island), B (mainland), C (coinfection with *Salmonella*–microfilariae; green dotted line), D (minimal infections), E (coinfection with saprophytic fungi and other organisms) and F (coinfection with other organisms but not with saprophytic fungi). *Salmonella*–microfilariae negative animals (red dotted line).

**Fig 10:**
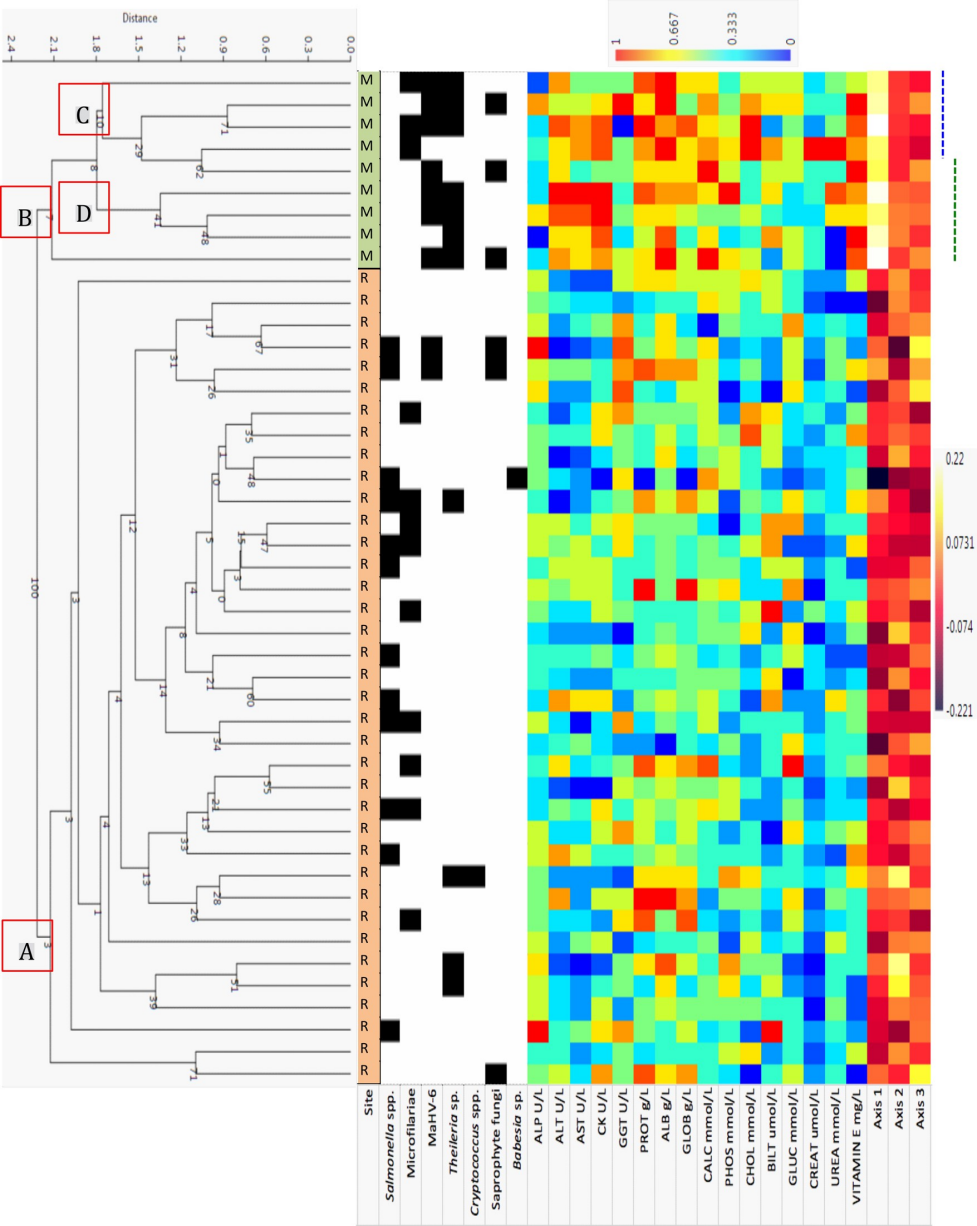
Cluster analysis and patterns of 16 BLC analytes of 47 animals from Rottnest Island (R) and 9 animals from the mainland (M) that were tested for seven infectious agents (*Salmonella* spp., microfilariae, MaHV-6, *Theileria* spp., *Cryptococcus* spp., saprophyte fungi and *Babesia* spp. Trypanosomes were not included as there were no positive animals). Clusters A (Rottnest Island), B (mainland), C (microfilariae-positive and coinfection with MaHV-6, *Theileria* spp., and saprophyte fungi), and D (microfilariae-negative and coinfection with MaHV-6, *Theileria* spp. and saprophyte fungi). General patterns in axes 1, 2, and 3 combined showed differences in microfilariae-positive animals (blue dotted line), and microfilariae-negative animals (green dotted line).

For the subset of animals tested for IA, cluster analysis revealed distinctions between mainland and Rottnest Island animals for both HMT and BLC profiles (Clusters A and B in both Figs 9 and 10). The generally higher BLC analyte concentrations are evident as warmer colours on the heatmap (Fig 10). For the Rottnest Island animals, two clusters were evident in the HMT dendrogram (Fig 9), with distinct differences in HMT profiles for animals testing positive for *Salmonella* and microfilariae (cluster C with generally lower values across all HMT analytes; evident as cooler colours in the heat map) and those that tested negative (cluster D; warmer colours in the heat map).

### Reference intervals

No animal met the criteria to be excluded from the dataset that was used to generate the reference intervals. Given the significant effect of *site* (results presented above) on the HMT and BLC profiles of quokkas, reference intervals are presented for mainland (Table 6) and Rottnest Island (Table 7) animals separately.

**Table 6:** Haematology and blood chemistry reference intervals for anaesthetised free-ranging quokkas sampled on the mainland of Western Australia between September 2010 and July 2011. Intervals calculated as being less than zero were set to zero (bold). Abbreviations and units as per Table 1.

							Reference Intervals ^a^ ^b^ ^c^		Confidence Intervals ^d^			
Analyte	*n*	Mean ^e^	Median ^f^	SD ^f^	Min. ^f^	Max. ^f^	Lower limit	Upper limit	2.50%	5%	90%	97.50%
**a) Haematology (HMT), *n* = 32–36**												
RBC	36	7.28	7.2	1.03	5.29	10.4	5.43	9.51	5.06	5.8	8.87	10.3
HGB	36	132	130	15.6	106	175	98.9	162	91.8	106	153	172
PCV	35	40.6	40	4.1	33	52	34	50.5	32.9	35	47.6	53.9
CHCM	36	326	318	28	290	391	261	375	250	277	356	391
MCV	36	59.8	59.7	3.24	54	66.3	53.8	66.9	52.8	55	65.2	68.8
Platelet	-	-	-	-	-	-	-	-	-	-	-	-
WBC	36	4.79	4.64	1.43	2.45	7.8	2.43	8.16	2.12	2.9	7.2	9.16
NEU	33	2.95	2.91	1.54	0.65	6.19	0.41	6.68	0.15	0.9	5.73	7.66
LYMPH	33	1.66	1.37	1.2	0.45	4.75	0.41	4.88	0.34	0.6	3.51	6.39
MONO	32	0.07	0.07	0.05	0	0.16	**0.00**	0.18	**0.00**	0.00	0.15	0.21
EOS	32	0.11	0.08	0.11	0	0.37	**0.00**	0.38	**0.00**	**0.00**	0.28	0.5
BASO	33	0.03	0.02	0.03	0	0.11	**0.00**	0.14	**0.00**	**0.00**	0.08	0.25
**b) Blood chemistry (BLC), *n* = 24–37**												
CK	37	7,674	5,858	7,071	397	20,000	229	32,034	71.9	629	24,127	41,123
ALT	34	413	405	107	181	692	218	654	183	263	580	733
ALP	29	2,996	985	4,123	251	13,580	185	27,954	132	269	10,825	72,737
AST	34	175	139	119	57	448	48.6	544	42.6	58.3	392	709
GGT	24	19.9	16.1	10.6	8	44	6.7	48	5.8	8.5	36.6	60.1
TP	37	63.8	63.8	4.63	53.7	71.9	53.8	72.6	51.1	56	70.7	74.5
ALB	37	39.1	39	2.38	34.6	44.2	34.7	44.4	33.9	36	43	45.9
GLOB	37	24.7	24.6	3.62	17.6	33.1	18.1	32.8	16.9	20	30.7	34.9
GLU	37	5.45	5.1	2.34	2	12.6	1.95	10.9	1.59	2.5	9.44	12.9
CHOL	37	2.99	2.92	0.72	1.8	4.4	1.7	4.6	1.5	1.9	4.21	4.99
BILT	35	5.04	5.1	1.91	0.9	8	0.93	8.81	0.83	2.1	8.06	9.55
UREA	36	9.16	8.39	3.9	3.8	20.2	3.78	19.2	3.2	4.5	15.8	22.8
CREAT	36	82.3	79.2	16.5	60	142	60.8	131	57.9	65	113	158
PHOS	37	1.68	1.56	0.72	0.7	3.5	0.62	3.42	0.52	0.8	2.91	3.97
CALC	37	2.48	2.46	0.16	2.17	2.9	2.21	2.83	2.16	2.3	2.73	2.95
Vit. E	32	9.92	9.77	2.85	5.65	15.1	5.23	16.5	4.67	6	14.6	18.3

Negative values of confidence intervals were interpreted as zero. Erythrocyte variables obtained with ADVIA® 120. WBC values obtained after correction with nucleated red blood cells. Polymorphonuclear cell values obtained with manual differential count.

^a^ RI computed using the robust method of the untransformed data

^b^ RI computed using the standard method of the Box-Cox transformed data

^c^ Reference intervals computed using the robust method of the Box-Cox transformed data,

^d^ Confidence intervals were computed using a bootstrap method (when 20 <n< 120)

^e^ for the standard method of the untransformed data

^f^ for the robust method of the untransformed data

† Insufficient sample size: 90% or more of blood smears were positive to platelet clumps

**Table 7:** Haematology and blood chemistry reference intervals for anaesthetised free-ranging quokkas sampled on Rottnest Island (RI) between March and December 2011. Intervals calculated as being less than zero were set to zero (bold). Abbreviations and units as per Table 1.

							Reference Intervals ^a^		Confidence Intervals ^b^			
Analyte	*n*	Mean ^c^	Median ^d^	SD ^d^	Min. ^d^	Max. ^d^	Lower limit	Upper limit	2.5%	5%	90%	97.5%
**a) Haematology (HMT), n = 40–113**												
RBC	113	5.83	5.81	0.86	4.14	8.51	4.26	7.69	4.14	4.56	7.46	8.51
HGB	113	108	108	15.1	53	149	79	141	53	87	131	149
PCV	101	33.6	34	4.5	20	47	23.1	43.9	20	27.1	40.9	47
CHCM	112	307	306	14.8	281	342	284	339	281	287	336	342
MCV	113	61	60.5	3.71	52.7	71.2	53	70	52.7	55.4	67.1	71.2
Platelet	40	501	493	110	322	813	322	810	322	336	703	813
WBC	113	4.64	4.39	1.65	1.75	9.31	2.03	8.55	1.75	2.29	7.82	9.31
NEU	106	2.09	2	0.73	0.82	4.65	0.93	4.01	0.82	1.05	3.38	4.65
LYMPH	108	1.99	1.76	1.09	0.59	5.36	0.62	5.12	0.59	0.67	4.16	5.36
MONO	106	0.07	0.06	0.06	0.00	0.26	**0.00**	0.25	**0.00**	**0.00**	0.18	0.26
EOS	107	0.36	0.28	0.31	0.00	1.46	0.03	1.39	**0.00**	0.06	1.01	1.46
BASO	105	0.01	0.00	0.02	0.00	0.09	**0.00**	0.07	**0.00**	**0.00**	0.06	0.09
**b) Blood chemistry (BLC), n = 83–111**												
CK	108	754	433	700	138	3,240	153.7	3,045	138	182.8	2,251	3,240
ALT	110	215	201	55.3	132	437	147	384	132	152	324	437
ALP	103	5,757	5,620	3,064	1,387	17,880	1,517	13,272	1,387	1,792	10,208	17,880
AST	107	45.1	40	17.7	13	108	25	97.2	13	26	84	108
GGT	83	17.8	17	6.2	8	41	8.1	30	8	10	28.9	41
TP	111	60.2	59.8	4.62	49.5	72.3	50.6	70.3	49.5	53.3	68.8	72.3
ALB	111	36.2	36.3	1.92	30	41.1	31.9	39.8	30	32.8	39	41.1
GLOB	111	24	23.5	3.78	14.4	36	15.7	32.4	14.4	18.6	30.7	36
GLU	111	4.15	3.70	2.18	0.6	12.5	0.68	9.94	0.60	1.06	7.94	12.5
CHOL	111	2.83	2.80	0.54	1.5	4	1.88	3.82	1.50	2.1	3.70	4
BILT	110	4.28	4	1.72	1	9.4	1.82	9	1	2	7.79	9.4
UREA	110	6.90	6.73	1.47	4.2	12.3	4.28	10.4	4.20	4.76	9.38	12.3
CREAT	110	71.3	68.7	15.6	41	112	47	110	41	49.6	104	112
PHOS	111	1.20	1.20	0.46	0.4	2.8	0.48	2.34	0.4	0.6	1.94	2.80
CALC	111	2.19	2.18	0.19	1.63	2.69	1.8	2.64	1.63	1.91	2.51	2.69
Vit. E	108	6.55	6.04	1.81	3.84	10.9	4.03	10.5	3.84	4.18	9.71	10.9

Negative values of confidence intervals were interpreted as zero. Erythrocyte variables obtained with ADVIA® 120. WBC values are those after correction with nucleated red blood cells. Polymorphonuclear cell values are those of the manual differential count.

^a^ reference intervals were computed using the non-parametric method (when n≥ 40)

^b^ confidence intervals were computed using a bootstrap method (when 20 < n < 120)

^c^ for the standard method of the untransformed data

^d^ for the robust method of the untransformed data

## Discussion

This report defines a set of haematology and blood chemistry reference intervals that can be used for the health management of free-ranging Rottnest Island and mainland quokkas. We found no sex differences in any of our analyses, but distinguished effects of site (Rottnest Island or mainland), sample time, as well as the presence of a suite of infectious agents on these blood parameters. While all of the animals sampled appeared healthy these differences reveal subtle influences that are revealed in their blood profiles.

### Site effects

Overall, site had the strongest influence on blood analytes with Rottnest Island and mainland animals presenting as significantly different subpopulations in terms of their HMT and BLC. In another study, Clark and Spencer [6] did not find any differences in the HMT of quokkas from the mainland compared to those from Bald Island. Bald Island exists in a cooler and wetter climatic zone compared to Rottnest Island [43] and is rarely visited by people [44]. Moreover, the total sample size in their study was quite small (Bald Island, n = 7; and mainland, n = 5). In contrast, others have demonstrated significant differences in HMT parameters between subpopulations of other free-ranging Australian marsupials e.g. western ringtail possums (*Pseudocheirus occidentalis*) [45], woylies (*Bettongia penicillata ogilbyi*) [46], and euros (*Macropus robustus*) [47].

In our study, mainland animals were held in traps for longer periods of time than their Rottnest Island counterparts and so may have been more stressed at the time of blood collection. A "stress leukogram" can be a response to stress [48–52] and the animals in this study from the mainland did have higher WBC, NEUT and BASO. More generalised increases in blood parameters can be due to dehydration (haemoconcentration) [29] but we did not appreciate any differences in the hydration status of the groups of animals following a clinical examination and not all blood analytes were higher in the Rottnest Island cohort. Other studies have suggested that the differences between blood analytes and site could be due to an interplay of factors such as site, climate, nutrition, social structure, age, the presence of infectious agents, and/or the presence of predators [3, 5, 29, 45–47, 53, 54].

Mean values for the leukogram component of the Rottnest Island animals were generally lower than values reported previously for captive quokkas [11, 13, 14]. For example, WBC and NEUT were approximately two-fold higher, and LYMPH averages were 3–6-fold higher, in reports of captive quokkas compared to what we found in free-ranging Rottnest Island quokkas. Leukocyte counts are affected by multiple factors [29], with lymphocytosis known to be a feature in many different infectious, neoplastic and hormonal conditions. Further research will be necessary to investigate this finding.

Mainland quokkas had higher concentrations of ALT, AST, ALP, CK and vitamin E than Rottnest Island quokkas. Although higher levels of the aminotransferases, AST and ALT, in conjunction with higher levels of ALP and GGT may indicate hepatic injury and insufficiency [55], there were no other blood chemistry results (e.g. low GLUC, high BILT, increased WBC) or clinical signs (e.g. ascites, icterus) that were consistent with liver disease. A more plausible explanation for these differences is the longer period of time that mainland animals were held in traps compared to their Rottnest Island counterparts. The higher levels of AST and CK may therefore have originated from muscle, and this has been suspected to be the cause of elevations in these enzymes in other studies [45, 56]. Elevations of ALT have also been associated with muscle injury [55]. Very high levels of ALP in quokkas from Rottnest Island were observed that far exceeded all other values for ALP reported in macropods [4, 5, 14]. The reason for this high value is unclear but future studies should consider searching for the corticosteroid-based isoform of ALP that has previously been described only in dogs [57]. Given these elevations were seen in adult animals, an osteoblastic origin of ALP [4, 5, 56] seems to be an unlikely source. Equally, it is doubtful that cholestasis explains the high levels of ALP seen given GGT was within the range reported for other apparently healthy macropod species (i.e. *M*. *giganteus*, *M*. *fuliginosus*) [14].

Blood chemistry analytes in quokkas were generally similar to previously reported data for other free-ranging [4, 5, 58] and captive [14] macropods. Our data differed considerably from the only published BLC data for quokkas [15], which comes from captive individuals. Both subpopulations (Rottnest Island and mainland) had higher values for ALP, ALT, AST, CK, BILT, PROT, UREA, and GLOB, sometimes by two-fold or more; and lower values (though not as different), for GLUC, CALC, ALB and CHOL compared with these published values. Differences in BLC profiles between free-ranging and captive subpopulations are not uncommon. This has been reported in other marsupial species, e.g. western ringtail possum (*Pseudocheirus occidentalis*) [45], Gilbert’s potoroo (*Potorous gilbertii*) [56], and western barred bandicoot (*Perameles bougainville*) [59], and the differences attributed to nutritional factors or handling stress (for CK and AST in particular). Published data are often obtained from the International Species Information System (ISIS) where sample sizes are often limited to a few animals, meaning that differences should be interpreted cautiously.

### Associations with sample time

Sample time had an effect on HMT, BLC and PBCM in free-ranging quokkas on Rottnest Island. The island has limited standing water and there is little moisture in vegetation over summer; this aridity causes nutritional deficiencies, starvation and dehydration in the quokka population [60]. There is a mass population crash on the island each year [61, 62], with most deaths in early autumn, when animals surviving low nutrition and dehydration over summer, weakened by loss of body condition, succumb to the first cold/wet nights [63]. We were unable to link these changes in the environment to the changes in the analyte concentrations seen across the sample times. Other studies observed a seasonal anaemia in Rottnest Island quokkas [8, 10] and it was thought that this was due to low dietary protein. In other macropods, anaemia has been associated with nutritional deficiencies [10], nematode infestation [64] and unknown causes [65]. Although our sample times corresponded to the seasons of the year, our results should not be used to conclude that there was a seasonal effect. To investigate the seasonality of an effect, each season needs to be sampled across multiple years. In our study, each season was only sampled once.

### Associations with potential infectious agents (IA)

Infectious agents were detected in 24 out of 58 quokkas tested (41%) and for all 24 of these quokkas, more than one IA was detected. Further, all eight of the IA that were tested for (*Salmonella*, microfilariae, *Macropod herpesvirus-6* [MaHV-6], *Theileria* spp., *Cryptococcus* spp., other saprophytic fungi, *Babesia* spp. and trypanosomes) were detected in this study. This allowed meaningful comparisons to be made between the blood analytes measured (HMT, BLC and PBCM) and the presence (or absence) of these IAs. The intended goal was not to explain the mechanisms responsible for such differences (and therefore to infer causality), but simply to describe possible patterns.

Infection with the microfilariae *Breinlia macropi* has been previously reported in quokkas [66, 67]. The presence of microfilariae has been considered to be a contributing factor in anaemic processes due to intravascular haemolysis as a result of destructive motility [68–71]. However, we did not observe significant differences in the PCV or RBC of microfilariae-positive or -negative animals

Of all the infections we detected in quokkas, the most common association with blood analytes was with mixed infections with *Salmonella* and microfilariae (seen only in Rottnest Island animals) or MaHV-6 and *Theileria* spp. (seen only in mainland animals).

For the animals infected with MaHV-6 and *Theileria* spp., lower BLC was observed in animals that were coinfected with microfilariae. The mechanism by which this might occur is unknown. We did not identify other significant coinfections but many of these other IAs were seldom detected.

Coinfections with bacteria, viruses, helminths, and protozoa have previously been reported (mostly studied in pairs), and both synergistic and antagonistic interactions have been observed with various effects on the host [72]. In helminth and bacterial coinfections, helminths have been reported to facilitate and enhance the bacterial component e.g. filarial and *M*. *tuberculosis* coinfection [73] and nematodes and *Salmonella enterica* ser. Typhimurium coinfection [74]. Similarly, coinfection with gammaherpesviruses (the genus that MaHV-6 belongs to) and helminths, have shown that the latter can induce viral reactivation if T helper cells Type 2 inflammation is present [75]. Helminths and protozoa have been reported to have complex interactions in the coinfected host, both synergistic [76] and antagonistic [77]. Viral and protozoal mixed infections have resulted in reduced body condition and higher mortality rates [78–80]. Of course, these micro-organisms are extraordinarily diverse and their interactions will invariably be dependent on microbe, host and environmental factors [81]. Further, much of the earlier literature stems from investigations of disease, and it is expected that many more subtle and asymptomatic interactions between the organism and the host, such as our study, will emerge. It should not be automatically assumed that the presence of an infectious agent will always have an adverse impact on the host. For example, studies in animal models have shown that latent infections of gammaherpesviruses can lead to an enhanced immune state that protects the host against other challenges [82].

### Vitamin E

The reference interval for vitamin E in the plasma of quokkas has not been determined. This is surprising considering quokkas have been used as a model for muscle dystrophy of nutritional origin [83–87]. These studies showed that the condition was not only triggered by vitamin E (alpha-tocopherol) deficiency [83] but also by small enclosures [85], and that clinical signs of muscle dystrophy such as weight loss, progressive wasting of hindlimb muscles and paralysis, could be reversed through the supplementation of vitamin E at a daily dose of 200–600 mg [84, 87]. In our study, the median and mean concentrations of vitamin E in the plasma of a small sample of captive quokkas (n = 8) from Perth Zoo was significantly lower than the median and mean values for free-ranging Rottnest Island quokkas (during winter). Published values for quokkas from Taronga Zoo (2.9–3.2 mg/L; n = 5) and Melbourne Zoo (2.0–2.6 mg/L; n = 4) are similarly low [14]. Differences between free-ranging and captive quokkas is likely to reflect content of vitamin E in their available diets; although the diets of Rottnest Island and mainland quokkas have been identified [88, 89], the chemical composition of these diets has not been determined to allow comparison with a manufactured diet. Our data suggests that captive individuals are not being adequately supplemented with vitamin E.

### Atypical lymphocyte morphologies

Atypical lymphocyte morphologies were detected in 23 of 108 Rottnest Island animals but not on animals from the mainland. These cells were thought to be Flower cells (of lymphocyte lineage), due to the appearance of a granular cytoplasm, coarsely clumped chromatin, and their smaller nucleus:cytoplasm ratio, when compared to MONO, NEUT and EOS. Surface markers and special stains would have facilitated the differentiation of these cells from other haematopoietic lineages [51], but these resources were not available to this project. If not Flower cells, these cells may have been lymphocytes that had lost their nuclear integrity due to a neoplastic alteration. Pleomorphic lymphocytes with a nucleus that varies in shape from a single indentation to a complex convoluted appearance are often observed in lymphoproliferative disorders in other species [51]. Irrespective of their identity, to the best of our knowledge, these cells have not been reported before in quokkas, or any other macropod species.

Proliferative disorders of the hematopoietic nature have been reported in a number of other mammalian species [90–92], including macropods [14, 90]; however, leukocyte morphology was not reliably recorded. Of note is that lymphocytes with similar atypical morphology have been observed in asymptomatic human carriers of human T-cell leukaemia virus type 1 (HTLV-1, a deltaretrovirus) [93]. To our knowledge, these cells have not been reported in people uninfected with HTLV-1. Flower cells have been observed in 7% of HTLV-1 carriers [94], and in more than 50% of patients with adult T-cell leukaemia/lymphoma (ATL) [95]. Some authors consider that the presence of these cells (i.e. Flower cells) in asymptomatic carriers of HTLV-1 is a risk factor for developing ATL [96, 97], while others believe these cells represent a preleukaemic event [98]. More recently, Flower cells have been recorded in mice five months after being infected with sub-lethally irradiated HTLV-1 producing cells [99]. Testing quokka blood with these atypical lymphocytes for the presence of deltaretroviruses should provide further insight into the significance of these cells.

### Limitations of this study

Some of the trapping and handling procedures were not within our control, and therefore the differences in animal holding time between Rottnest Island and mainland populations could not be standardised. As described above, some influence of this difference may be reflected in the blood profiles of animals sampled. We also had little control over the time of year that samples were collected from mainland animals, and consequently could not take this into account in analyses. Further sampling, now that we are aware that time of sampling influenced blood profiles, could benefit understanding.

## Conclusions

This study provides the most comprehensive haematology and blood chemistry reference intervals for free-ranging quokkas. We have utilised novel methods by which the complex interactions between blood analyses and other factors (sample time, sex, site and infectious agents) can be explored. Our data have shown that there are significant differences between the HMT and BLC profiles of the subpopulations studied (Rottnest Island and mainland), and highlights the importance of establishing reference intervals not just for the species as a whole but for individual subpopulations, given the key differences in their respective environments and the infectious agents they harbour. We echo other voices advocating that disease and health surveillance should be an ongoing effort in native species management plans. This would allow for the development of contingency plans that would assist relevant management bodies to respond rapidly to new disease events.

## Supporting information

S1 File Diet of captive quokkas from the Perth Zoo. (DOCX)

S1 Table Semi quantitative body condition scores used in quokkas. (DOCX)
