# Supplementary material for: Haematology and blood chemistry in free-ranging quokkas (Setonix brachyurus): Reference intervals and assessing the effects of site, sampling time, and infectious agents
Source: PLoS One. 2020 Sep 17;15(9):e0239060. doi: 10.1371/journal.pone.0239060 (PMC7498088; doi:10.1371/journal.pone.0239060)
Supplement: S1 File — (DOCX) [file pone.0239060.s001.docx]

The diet provided to these animals included manufactured macropod pellets (unknown concentration of vitamin E), sprinkled with Value Plus Vitamin E Powder® (Value Plus Animal Health Care Products Pty Ltd, NSW Australia at a dose of 1 g/animal/day, i.e. 52 mg of d-alpha tocopheryl acid succinate); quokka cubes (Specialty Feeds, WA Australia) containing 1,600 mg of alpha tocopherol acetate per 1 kg of product; Olsson's 007 Pressed Nutritional Mineral Block® (Olsson’s, WA Australia; vitamin E concentration not available); browse species (e.g. *Acacia*, *Brachychiton*, and *Ficus*); and lucerne hay (alfalfa).
