# Supplementary material for: Haematology and blood chemistry in free-ranging quokkas (Setonix brachyurus): Reference intervals and assessing the effects of site, sampling time, and infectious agents
Source: PLoS One. 2020 Sep 17;15(9):e0239060. doi: 10.1371/journal.pone.0239060 (PMC7498088; doi:10.1371/journal.pone.0239060)
Supplement: S1 Table — (DOCX) [file pone.0239060.s002.docx]

**S1 Table.** Semi quantitative body condition scores used in quokkas.

| Body condition scores | Description | Features observed that defined each score |
| --- | --- | --- |
| 1 | Emaciation | Scapular spine and dorsolateral and lateral processes of the first two coccygeal vertebrae are prominent. There is an obvious concavity of skin and muscle around these vertebrae). |
| 2 | Poor | Scapular spine and dorsolateral and lateral processes of the first two coccygeal vertebrae are still palpable and visible. The concavity of skin and muscle around them is less. |
| 3 | Optimal | Scapular spine and dorsolateral and lateral processes of the first two coccygeal vertebrae are barely palpable. Concave appearance of the skin and muscle around the bony prominence is not present. |
| 4 | Overweight | Scapular spine and dorsolateral and lateral processes of the first two coccygeal vertebrae are not palpable. There is a slight convex appearance of the skin and muscle around the bony prominence. |
| 5 | Obese | Scapular spine and dorsolateral and lateral processes of the first two coccygeal vertebrae are not palpable. Marked convex appearance of the skin and muscle around the bony prominence. |
